# Using Co-Design to Adapt a Digital Parenting Program for Parents Seeking Mental Health Support

**DOI:** 10.3390/children13010129

**Published:** 2026-01-15

**Authors:** Meg Louise Bennett, Ling Wu, Joshua Paolo Seguin, Patrick Olivier, Andrea Reupert, Anthony F. Jorm, Sylvia Grant, Helen Vaxevanis, Mingye Li, Jue Xie, Marie Bee Hui Yap

**Affiliations:** 1 Turner Institute for Brain and Mental Health, School of Psychological Sciences, Monash University, Clayton, VIC 3800, Australia; meg.bennett1@monash.edu; 2 Action Lab, Faculty of Information Technology, Monash University, Clayton, VIC 3800, Australia; 3 School of Educational Psychology and Counselling, Faculty of Education, Monash University, Clayton, VIC 3800, Australia; 4 Melbourne School of Population and Global Health, The University of Melbourne, Parkville, VIC 3010, Australia; 5 IPC Health, P.O. Box 171, Deer Park, VIC 3023, Australia

**Keywords:** co-design, parenting, parental mental health, service design, parenting intervention, digital health, mental health services, clinician, acceptability

## Abstract

**Background/Objectives**: Parental mental health challenges are associated with parenting difficulties and child mental health issues. Parenting interventions can support families; however, parents with mental health challenges face barriers to accessing parenting support, which is not consistently offered within adult mental health settings. Embedding technology-assisted parenting programs into these settings could provide accessible, holistic support. Partners in Parenting Kids (PiP Kids) is a digital parenting program designed to prevent child anxiety and depression, yet its suitability for parents with mental health challenges and fit within mental health services remains unclear. This study aimed to co-design and adapt PiP Kids for future implementation in an Australian adult mental health service. **Methods**: Parents who recently sought mental health support (*n* = 8) and service providers (*n* = 7) participated in co-design workshops to explore needs and preferences for a technology-assisted parenting program and iteratively develop a prototype. Parents (*n* = 3) trialled the online component of the prototype and participated in qualitative interviews to assess acceptability. **Results**: The adapted clinician-supported program was designed to facilitate (1) parent and clinician readiness for parenting support; (2) emotional and social support for parents and clinicians; (3) practical, personalised parenting knowledge; (4) parent-led empowerment; and (5) accessible, integrated support. Prototype clinician training was developed to strengthen the clinician-support component. Parents indicated initial acceptability of the online prototype while reiterating the value of including face-to-face support. **Conclusions**: This study co-designed an online, clinician-supported parenting program for future embedding within adult mental health settings. The findings highlight key considerations for developing and implementing technology-assisted interventions that promote family-focused care for parents seeking mental health support.

## 1. Introduction

Children of parents experiencing mental health challenges, such as anxiety and depression, are up to twice as likely to experience their own mental health difficulties, especially if families do not have adequate support [1,2]. Parental mental health challenges are linked with an increased likelihood of parenting difficulties, including reduced warmth and increased overcontrolling, harsh, or inconsistent parenting [3,4,5,6,7]. These parenting difficulties have been identified as a key mechanism in the intergenerational transmission of mental health issues [8,9,10] and can worsen parents’ mental health, creating a vicious cycle of distress within families [11,12]. Fortunately, parenting is a modifiable factor, with evidence-based parenting interventions shown to improve parenting and child outcomes for parents with and without mental health challenges [13,14,15]. However, access to parenting intervention for these parents can be limited, and they are not consistently offered parenting support within mental health treatment pathways [16]. This is a missed opportunity given the well-established interrelationships between parenting, parental mental health, and child outcomes [17,18].

Integrating parenting support into parent mental health care can offer holistic, family-focused care for families, while enhancing the recovery of parents [11,19]. Research shows that for parents with mental health issues, interventions targeting both parental mental health and parenting yield more wide-ranging benefits across parent symptoms, parenting, and child outcomes compared to those targeting either issue alone [19,20]. Adult mental health services are well placed to provide a combined approach, as they can extend their support of parent mental health to address parenting as one aspect of the parent’s identity, functioning, and wellbeing [21,22]. There are several face-to-face interventions and initiatives that integrate parenting support within adult mental health services [21,23,24,25], for example, Let’s Talk about Children [26,27] and the ParentingWell practice approach [28,29]. These have been found to be acceptable among parents and clinicians and to be effective in improving parent mental health, parenting, and child outcomes, as well as clinician confidence and skills in supporting parenting [27,30]. Despite this, implementation of family-focused support into mental health settings can be hindered by service barriers, including a lack of resources, training, time, and clinician confidence to deliver parenting intervention alongside mental health care [31,32]. Additionally, parents experiencing mental health challenges often face barriers to accessing in-person support, such as stigma, competing priorities, fluctuating mood, low energy, childcare demands, and transportation difficulties [33,34,35].

One promising approach is implementing technology-assisted parenting programs in existing mental health services, where parenting support is provided partially or entirely via technology [36]. Technology has the potential to provide parents with accessible, flexible parenting support alongside mental health treatment, by minimising barriers such as stigma, lack of time, and scheduling demands [37,38]. By reducing reliance on clinicians, this approach can also decrease service burden and costs and support feasible long-term implementation in resource-limited adult mental health services [39,40]. Importantly, evidence is emerging that technology-assisted parenting programs are acceptable and effective in improving parenting and child outcomes among parents with mental health challenges [41,42,43]. However, to our knowledge these programs have not yet been embedded within adult mental health settings.

Partners in Parenting Kids (PiP Kids; formerly known as Parenting Resilient Kids or PaRK) is a digital parenting program that may be well-suited for integration. PiP Kids is a fully online program that provides parents with evidence-based parenting strategies to reduce the risk of anxiety and depression in their primary school-aged children [44,45]. The original program comprises a self-assessment of parenting practices, a tailored feedback report, and self-guided online modules [44]. In a randomised controlled trial, parents who received PiP Kids showed greater improvements in parenting behaviours compared to those receiving a factsheet intervention [46]. This evidence of efficacy, together with the successful adaptation of PiP Kids to family services for parents of children experiencing adversity [47], suggests its potential for integration within adult mental health treatment.

Yet, it remains unclear how well a digital parenting program such as PiP Kids will fit into the existing clinician and service practices at adult mental health services. Additionally, parents with mental health challenges have unique parenting needs, including talking with children about parental mental health, managing their mental health symptoms alongside parenting, and navigating increased parenting stress [48,49,50]. These parents may also face unique barriers to engaging with parenting programs, such as debilitating symptoms, low literacy, socioeconomic disadvantage, and social isolation [34,35]. Generic or fully digital parenting programs such as PiP Kids may therefore not adequately address the specific needs or preferences of these parents [21,51]. This highlights the importance of adapting PiP Kids to ensure its acceptability and compatibility with existing service practices and family circumstances.

Co-design or collaboration with relevant stakeholders and end-users is an ideal approach for ensuring a thorough understanding of and tailoring to the needs of parents and existing health services [52]. Actively engaging parents in designing programs is needed to develop an intervention that meets their needs and preferences [53,54]. Similarly, service provider perspectives are crucial as they possess specialised knowledge of service practices, are instrumental in advocating for families, and may also be key end users involved in program delivery [47]. Research suggests that adapting programs to fit the context, procedures, and workload of the existing health service, while retaining evidence-based content, helps ensure the program is relevant and successfully integrated [29,35].

The aim of this study was therefore to co-design and adapt an evidence-based digital parenting program (PiP Kids) for future integrative implementation in an existing adult mental health service, to support parents seeking mental health care. Specifically, this study used co-design methods to (1) identify design considerations for integrating technology-assisted parenting programs by exploring the needs and preferences of parents with lived experience of seeking mental health support and service providers who support these parents. This study then aimed to (2) design, develop, and refine an adapted prototype parenting program, and (3) deliver the online component of the prototype program to parents, to understand its initial acceptability and identify what further adaptations are needed.

## 2. Materials and Methods

### 2.1. Study Setting

This study was conducted as part of a larger research program, in partnership with a major provider of community health services in Victoria, Australia. As this study specifically focuses on parents seeking mental health support, the mental health services within this community health organisation (including therapeutic counselling for individuals experiencing mild-to-severe mental health challenges, gambling, and alcohol and other drug use) were selected as the most appropriate setting for the study.

### 2.2. Recruitment

We aimed to recruit a small number of participants in this initial study to gain detailed insights into parent and service needs. Small sample sizes are generally considered acceptable in in-depth qualitative studies [55]. Co-developing and piloting an initial prototype on a small scale is considered a feasible and cost-effective way of designing and refining new interventions during the development stages [56]. Findings from these initial iterations can then be used to guide larger-scale iterations and eventual implementation.

#### 2.2.1. Service Providers

Service providers were clinicians and clinical leads employed at the collaborating mental health service, who spoke English and had experience providing mental health support to parents of children aged 5–11 years. All eligible service providers within the collaborating mental health service were invited to participate via an emailed flyer.

#### 2.2.2. Parents

All parent participants were parents or primary caregivers of children aged 5–11 years who spoke English and lived in Australia. Parents were either (1) currently seeking mental health support at the collaborating community health service or (2) had received psychological support for their mental health in the community within the past year. Recruitment occurred via two methods. First, clinicians at the collaborating mental health service distributed flyers to potentially eligible parent clients. Second, flyers were distributed via websites, social media, waiting rooms, and mailing lists of community organisations across Australia that support parents with mental health challenges. Although the study aimed to recruit across parents experiencing general mental health challenges, gambling, and alcohol and other drug use, the recruited parents all self-reported general mental health challenges such as anxiety, depression, or stress.

### 2.3. Study Design and Procedure

**Figure 1:**
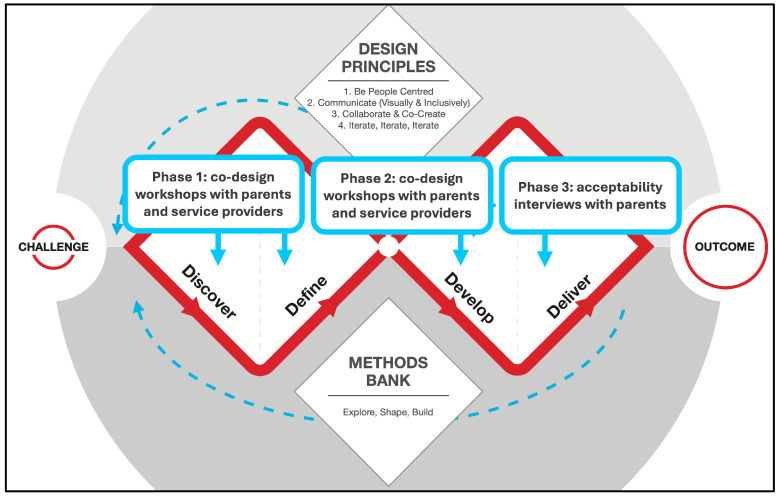
The Double Diamond design process, illustrating how the discover, define, develop, and deliver stages align with the phases of the current study, as indicated by the blue arrows.

The Double Diamond design process guided the co-design methods in this study [57]. Each diamond represents a process of examining an issue broadly followed by taking focused action. As shown in Figure 1, this study was conducted in three phases that align with the Double Diamond stages. Phase 1 involved workshops with parents and service providers to ‘discover’ needs and preferences and ‘define’ design considerations for integrating technology-assisted parenting programs into a mental health service. Phase 2 involved workshops with parents and service providers to ‘develop’ an initial prototype and validate and refine this design and the design considerations. Phase 3 involved ‘delivering’ the online component of the refined prototype to parents and conducting one-on-one interviews to explore initial acceptability and identify further adaptations for the next iteration. See Supplementary Table S1 for a summary of the aims and methods. Due to the iterative nature of the study, further methodological details for each phase are provided within their respective Methods Sections below, with each phase informed by findings from the previous one.

### 2.4. Ethical Considerations

All procedures were approved by the Monash University Human Research Ethics Committee (approval numbers #35059 and #44564). Participants were provided with a plain-language explanatory statement and the opportunity to ask questions prior to consenting. Parent participants were reimbursed with a $35 gift card for each hour spent engaging in a workshop or interview. Service provider participants were not financially compensated, as research activities were conducted during their working hours. For parents currently seeking support at the collaborating service, clinicians initially provided parent contact details to the research team. Once parents were recruited, all individual parent data were collected and stored securely by the research team and were not shared with the partner service, regardless of whether parents were currently seeking support from the collaborating service.

Co-design workshops were conducted separately for each participant group to create a safe environment for open discussion and minimise the influence of power imbalances. To ensure parent participant wellbeing, all workshops and interviews were facilitated by a provisional psychologist (the first author) with formal training in mental health, under the supervision of a registered psychologist. Regardless of whether parent participants demonstrated distress, the first author contacted them within 24 h of their workshop or interview to assess for distress and offer support and crisis resources.

### 2.5. Researcher Positionality and Reflexivity

The first author is a female clinical psychology PhD candidate and provisional psychologist with interests in mental health, parenting, and evidence-based intervention. Other co-authors involved in data interpretation included two academics with expertise in co-design and qualitative research and one professor who is a parent and psychologist with 14 years’ experience working with parents, young people, and service providers in co-designing and developing parenting interventions. The remaining co-authors who reviewed the findings and manuscript included professors with expertise in parental mental health, prevention and intervention in mental health, co-design and human–computer interaction, as well as service providers with expertise in clinical service delivery and management, several of whom have lived experience of parenting themselves. The first author facilitated all workshops and interviews. While the prompts used during these activities were designed to be open-ended, the first author’s choice of prompts and management of group dynamics may have influenced participant responses. Group workshops may have encouraged sharing of perspectives, whereas individual sessions may have encouraged more personal reflection. The first author kept a reflexive journal following each activity, documenting her reflections, including how facilitator choices may have influenced data. During analysis, a conscious effort was also made to ensure a balanced integration of perspectives across all three participant groups (parents, clinician, and clinical leads) when triangulating findings and identifying themes.

## 3. Phase 1 Methods and Results: Discover and Define Design Considerations

### 3.1. Phase 1 Methods

#### 3.1.1. Participants

Parent participants (*n* = 8) were all mothers aged 39–46 years (M = 41.1). One was currently seeking mental health support at the collaborating health service, and seven had recent lived experience of seeking mental health support in the community. Parents’ self-reported mental health challenges included high stress (*n* = 7), anxiety (*n* = 5), depression (*n* = 4), trauma (*n* = 3), and one each with psychosis, bipolar disorder, and grief. Parents’ reported cultural backgrounds included Australian/New Zealand (*n* = 4), European (*n* = 2), Pacific Islander (*n* = 2), and one each of East Asian, Middle Eastern, and South Asian.

Service provider participants included clinicians (*n* = 5) and clinical leads (*n* = 2), all of whom were women. Clinicians were aged 25–50 years (M = 37.8) and included counsellors (*n* = 2) and social workers (*n* = 3) who primarily worked with mild-to-severe mental health challenges (*n* = 4) or gambling concerns (*n* = 1). Clinicians’ reported cultural backgrounds included one each of Australian/New Zealand, European, Middle Eastern, Pacific Islander, South Asian, and Māori. Clinical leads were aged 52–59 years (M = 55.5) and included one team leader who was a psychologist and one service manager who was a social worker. Clinical leads reported their cultural backgrounds as one each of Australian/New Zealand and European. Three clinicians were also mothers with lived experience of depression and/or anxiety, with many of their responses combining their personal and professional perspectives. Several of these instances are highlighted in the results. Supplementary Table S2 details further demographic characteristics of the Phase 1 participants.

#### 3.1.2. Data Collection

Phase 1 workshops explored parent and service provider needs and preferences for any technology-assisted parenting program integrated into mental health care. Participants completed a brief demographic questionnaire using REDCap (Research Electronic Data Capture) [58,59] before taking part in workshops. Workshop focus and format were informed by relevant literature on co-design, parenting in the context of parental mental health challenges, parenting programs, program adaptation, real-world implementation, and clinician experiences and training. All workshops were conducted via Zoom, with audio and video recording. Each workshop lasted 2 h, including a 5–10 min break.

#### 3.1.3. Parent Workshops

**Figure 2:**
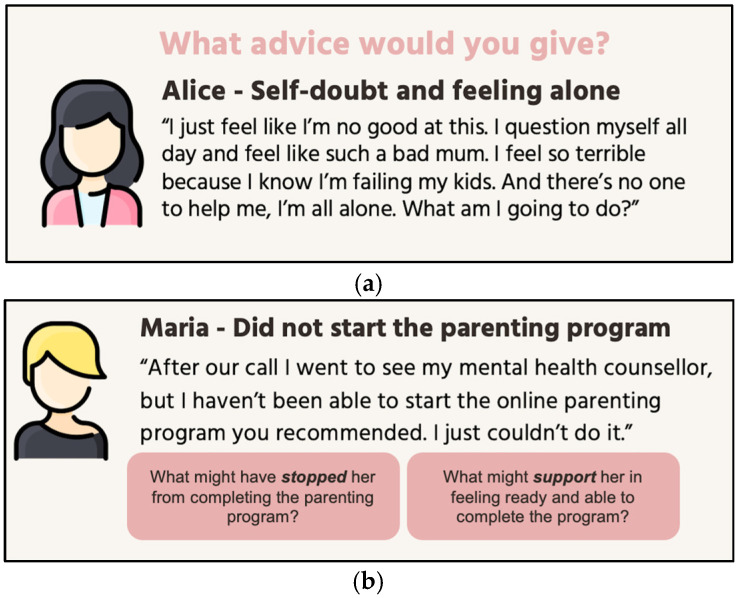
Examples of activities from the Phase 1 parent workshop demonstrating (**a**) an activity exploring strategies for supporting parents with parenting challenges; (**b**) an activity exploring barriers to engaging with parenting programs and strategies for support.

Each parent participated in one individual or group workshop during this phase. As parents with mental health challenges often describe feeling guilt, shame, and helplessness [60,61], this workshop used case-vignettes to create psychological distance where parents could draw on their lived experience reflectively and empathetically without being overwhelmed [62,63]. In the vignettes, parent participants role-played as helpline workers providing advice to the vignette parents. Activities explored challenges faced by parents in parenting, engaging with parenting programs, and applying parenting knowledge, as well as strategies to support parents in these areas. Figure 2 provides examples of activities from the parent workshop.

#### 3.1.4. Service Provider Workshops

**Figure 3:**
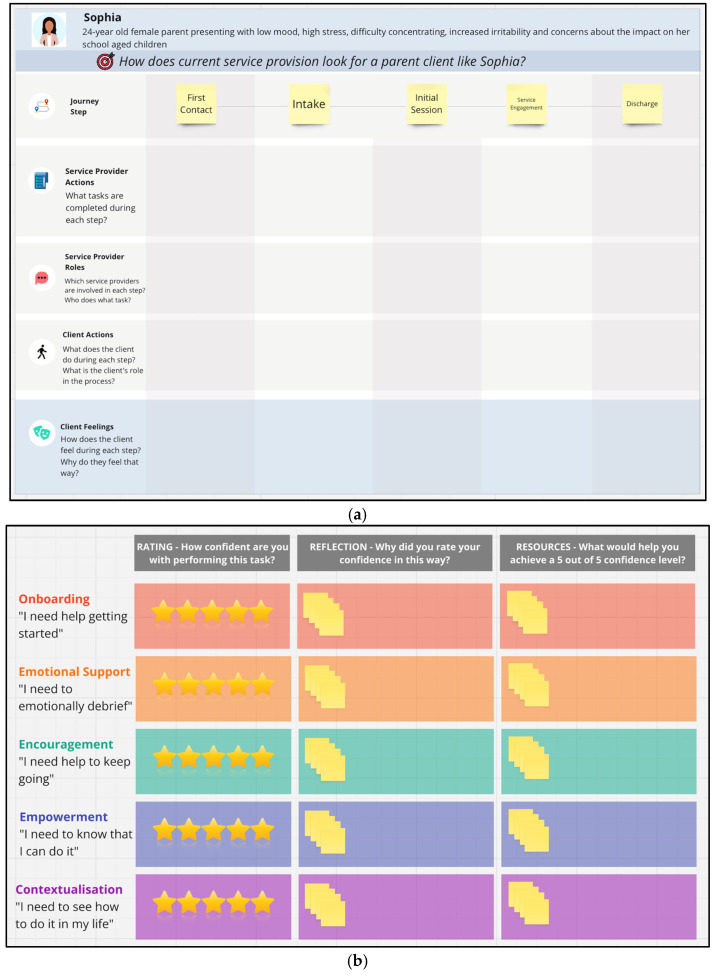
Examples of activities from Phase 1 service provider workshops demonstrating (**a**) an activity from the round-one workshops that mapped service provision and clinician roles within the collaborating mental health service; (**b**) an activity from the round-two workshops that explored clinician confidence in undertaking parenting support tasks and identified supports to strengthen this confidence. Yellow stars and sticky notes were used to record participant responses during the activities.

Service providers attended two rounds of workshops in this phase. The first round examined the service context, focusing on day-to-day service delivery for clinicians and on broader program integration and organisational resources for clinical leads. This round also explored general challenges faced by parents in applying parenting knowledge and opportunities and barriers within the service to support them. The second round of workshops built on findings from prior workshops by first exploring service provider feedback on emerging themes. Findings from the first round of workshops with parents and clinicians indicated a clear need for clinicians to support parents in engaging with any digital parenting program. Consequently, this round of workshops focused on how to enable clinicians to provide this support. For clinicians, this involved identifying the resources and support they would need, while for clinical leads the focus was on determining what organisational resources could be provided to address these needs. Figure 3 presents example activities from these workshops.

#### 3.1.5. Data Analysis

Recordings were transcribed verbatim by an automatic artificial intelligence transcription service (Descript version 2.10.0) and corrected by the research team. Transcripts were analysed with the support of NVivo qualitative software version 15.3.0, using Braun and Clarke’s reflexive thematic analysis approach [64,65]. Data were coded inductively as the purpose of this phase was to broadly explore the needs and preferences of parents and service providers. We followed the six-phase process of reflexive thematic analysis: (1) data familiarisation; (2) iterative data coding; (3) initial theme generation; (4) theme development and review; (5) refining, defining, and naming themes; and (6) writing up the findings [65,66]. The development and refinement of themes was carried out by the first author in collaboration with the broader research team.

### 3.2. Phase 1 Results

Participants described five design considerations for adapting the parenting program to address parent and service provider needs and preferences, as identified in the following themes: (1) building parent and clinician readiness for parenting support, (2) emotional and social support for parents and clinicians, (3) practical and personalised parenting knowledge, (4) parent-led empowerment, and (5) accessible and integrated support. Supplementary Table S3 presents each theme and subtheme with additional indicative quotes.

#### 3.2.1. Theme 1: Building Readiness for Parenting Support

Participants described foundational factors influencing parent and clinician readiness to engage in parenting-focused support. Readiness was seen to depend on the wellbeing and safety of parents and their family, clinician training, and both groups’ understanding of the purpose and value of the program.

**Parental wellbeing foundations**

All participants emphasised that parents’ mental health, safety, and physical wellbeing are prerequisites for their capacity to parent effectively and engage with parenting support. Parent self-care, breaks from parenting, and attention to physical health were highlighted as particularly important. Declining mental health, or risk concerns such as family conflict, suicide risk, or child protection, were seen as needing stabilisation prior to parenting support. Service providers similarly emphasised the service’s primary focus on parental mental health, with parenting considered a secondary focus when relevant to parent wellbeing.

“They would talk about the difficulties they have with their young kids… but the main focus… the reason they came to me was because of their own mental health. That was the primary issue.”

[Clinician 4]

**Program understanding and confidence**

Participants identified understanding the program and feeling confident about its value and use as essential prerequisites among parents and service providers. All groups highlighted their need for a detailed program introduction, including its purpose, content, benefits, and technology. Parents suggested a clinician could support their introduction and help overcome barriers such as program uncertainty, fear of change, and difficulty starting. Some clinicians expressed low confidence in parenting issues and requested refresher training on child development, parenting, and supporting parents.

“Not training on just the program but training on just working specifically with mums… low self-esteem, feelings of guilt, shame, all that stuff that comes along with being a mum.”

[Clinician 5]

Clinical leads proposed that clinicians already possess this knowledge and that training should primarily focus on the program and building clinicians’ confidence in applying their clinical skills to providing support on parenting.

#### 3.2.2. Theme 2: Emotional and Social Support

Emotional support and social and peer connection were seen as crucial for both parents and clinicians.

**Emotional support and validation**

Participants (including clinicians with lived experience) highlighted the need to provide parents with non-judgmental emotional support that avoids criticising them for their mental health difficulties. Normalisation and validation of parent experiences were emphasised to remind parents they are not alone, acknowledge struggles as common, and foster hope. Participants stressed the need for parents to emotionally debrief with support people who listened with empathy and understanding. Strong therapeutic relationships with counsellors or psychologists built on trust, continuity, and rapport were seen as essential for parents to safely engage in support.

“You don’t often share what’s in your mind for fear of judgment… and that is the independent source that is hopefully listening and not judging and giving empathy… Just someone to listen to you is extremely powerful.”

[Parent 6]

**Social and peer connection**

Social support was seen as vital for parents and clinicians. Participants noted that connecting parents to family experiences or peers with lived experience of mental health challenges fostered support, belonging, and joy, and reduced isolation and self-blame.

“It would be good for her to meet other mums… so you get to share experiences and… gets to hear that she’s not alone… everyone goes through that same feeling that she’s going through… just getting connected and being able to hear other mums, I think that would be good for her.”

[Parent 3]

These connections were seen to provide practical support, such as sharing strategies or help with housework and childcare, which was described as particularly crucial when parental mental health was poor. However, participants expressed how social connection, such as support groups, could have downsides, including parents feeling shame or anxiety. Clinicians stressed their need for working in specialised, collaborative teams of peers to enhance confidence, shared learning, and work quality.

“As a clinician and working in this particular area if it was kind of specific… working within a team where we’re all on the same page and we’re able to kind of help and support one another… that’s how I’d build on my confidence.”

[Clinician 1]

#### 3.2.3. Theme 3: Practical and Personalised Knowledge

Participants expressed that parents need program content that extends beyond information to provide practical and personalised guidance.

**Practical knowledge**

Participants, including clinicians drawing on personal experience, emphasised that parents would benefit from concrete examples of how to use strategies and plans, reminders, and goal setting to translate knowledge into action. Practicing skills through role-playing with others and trying strategies with children was seen as essential. Participants highlighted that parents need practical steps and support for problem-solving barriers. Parents and clinical leads suggested that this be supported by a clinician who could follow up on issues, answer questions, and help parents consolidate and implement strategies.

“They might have read something and they were like, ‘I’m not really understanding how to put this into practice… I’ve read it, but I’m not sure how to implement it’. So maybe the clinician can assist with that.”

[Clinical Lead 2]

**Personalised knowledge**

Participants clarified that knowledge is effective only when tailored and contextualised to each parent, as every family is different. All participants described personalisation as requiring understanding of each parent’s unique context, mental health challenges, and barriers, then adapting strategies to be relevant and useful. Parents suggested that clinicians could support this through recommending modules and customising strategies based on each parent’s changing needs.

“That kind of personalised process… to understand what a parenting program means to her… Or maybe… she doesn’t have the time, so maybe the counsellor can help her prioritise… that would be hard for [parent] to do on her own, and it’s probably not a component of the parenting program itself.”

[Parent 5]

Parents and service providers also highlighted the need for content that is culturally inclusive and specific to parenting with mental health challenges, to ensure parents can see their family context in the program.

#### 3.2.4. Theme 4: Parent-Led Empowerment

All participants emphasised the importance of supporting parent empowerment and autonomy through helping parents feel more capable, confident, and in control.

**Guided autonomy**

Participants identified autonomy as central to empowering parents in their mental health and parenting, whilst acknowledging that parents would need professionals to support this autonomy through guidance and encouragement. It was seen as essential that this support was collaborative to help parents feel in control and guided rather than directed. Online and clinician support was viewed as most powerful when it respected parents’ ideas and helped them reflect, build on existing knowledge, and generate their own solutions. Participants stressed the need for clinicians to adopt a parent-centred approach led by parent preferences and priorities, consistent with the service’s client-led model.

“Sometimes people, they know what’s the best to do, but… don’t have the capacity. So I definitely agree with… not to tell her what to do. I would like to ask her… what do you think we can do in this situation… what’s your suggestion?”

[Parent 4]

**Parent self-efficacy and self-worth**

All participants, including clinicians with lived experience, emphasised the need to support parent self-efficacy and self-esteem to counter feelings of guilt, shame, inadequacy, and self-blame. Encouraging parents to embrace ‘good enough’ parenting, set realistic goals, and approach tasks one step at a time were viewed as crucial for building self-worth and a sense of achievement. Participants described the value of reinforcing parents’ existing strengths and acknowledging incremental progress to boost parent confidence and motivation to keep going. Clinicians were identified as playing a key role in this.

“Helping the parents to look… at the positives first… working within the strength-based model. And helping parents to identify their own character strengths so they can use that to… build on their parenting.”

[Clinician 3]

#### 3.2.5. Theme 5: Accessible and Integrated Support

Parents and service providers emphasised that program delivery must be accessible, flexible, and seamlessly embedded into everyday life and clinical practice

**Low-burden accessibility**

All participants valued a simple, engaging, and adaptable program to maximise usability. A mix of written, visual, and interactive delivery was perceived as important for maintaining parent engagement across learning styles. Participants asked for easy-to-follow, plain-language content to accommodate parents with diverse literacy levels or reduced cognitive functioning. They also recommended minimising the burden for parents with limited time and fluctuating symptoms by dividing the program into short sections with breaks, providing reminders, and ensuring flexibility to access the program at any time of day and to pause and re-engage as needed.

“It can’t be too long… parents might have things they need to organise for the kids… Breaking it down into smaller portions so that it’s achievable… because they could be tired and they may not be able to concentrate for too long.”

[Parent 8]

Participants highlighted the importance of the program being low-cost and offering user-friendly technology accessible across devices, to suit parents with different incomes and technology resources, as well as parents and clinicians with varying digital skills.

**Seamless integration**

Participants highlighted that effective program delivery depends on seamless integration into routine clinical practice, reflecting their view that mental health and parenting are closely intertwined. A self-directed program complemented by clinician check-ins during existing sessions was suggested to minimise clinician burden, leverage existing clinician skills, and extend support beyond limited clinician contact.

“Can kind of check in with people during that session… if it’s within… that timeframe, yes. But outside of that, it might be a challenge because of the other demands of clients.”

[Clinician 1]

Service providers emphasised the need to use case-coordination, secondary consultation, or referrals to specialists when parents’ needs exceed expertise or service scope, which parents also valued. Clinician training was recommended to be brief and flexible, including ensuring staff can learn at their own pace, revisit content, and access optional extra resources to accommodate diverse needs and schedules. While clinicians suggested supervision from parenting specialists, clinical leads considered this infeasible and proposed that this support be provided by existing supervisors instead.

#### 3.2.6. Intervention Design

The research team synthesised the above findings and translated the design considerations into relevant design features for the intended adapted parenting program. Features were designed and refined iteratively, drawing on relevant literature, features from the original PiP Kids program, and multidisciplinary meetings with psychology and human–computer interaction researchers from the team. For example, the subtheme ‘Parent self-efficacy and self-worth’ was conceptualised as including strengths-based and encouraging language, achievable strategies, and progress indicators within each online module, among other features. All concretised features are presented in Supplementary Table S4, mapped to the theme and subtheme they were designed to address and the program component where they would be embedded. Broadly, the adapted parenting program, named ‘Partners in Parenting Kids Parental Mental Health (PiP Kids PMH; herein ‘the program’), is intended to include four key components and a clinician training package. See Table 1 for a description of the main components of the program.

## 4. Phase 2 Methods and Results: Develop and Validate the Initial Prototype Design

### 4.1. Phase 2 Methods

#### 4.1.1. Participants

Participants included the same parents (*n* = 8) and all service providers from Phase 1, except one social worker clinician who had left the collaborating service (*n* = 2 clinical leads; *n* = 4 clinicians).

#### 4.1.2. Data Collection

**Figure 4:**
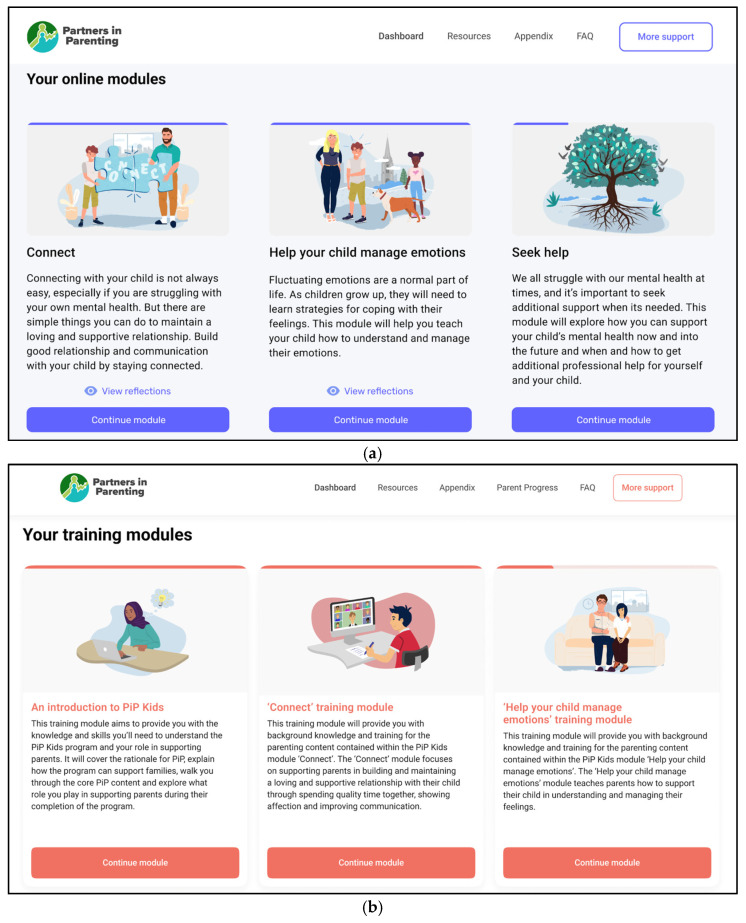
Dashboard pages including example modules from the online components of the initial prototype of the (**a**) parenting program and (**b**) associated clinician training modules, demonstrating the layout, wording, example topics, and functionality of the prototype.

Based on Phase 1 findings, the research team developed an initial prototype of the adapted parenting program and associated clinician training, each demonstrating the dashboard and two example modules. Figure 4 shows the online dashboard pages from the initial parenting program prototype and the accompanying clinician training modules. Phase 2 workshops aimed to validate and refine the program design with parents and service providers. Each parent and service provider participated in one workshop during this phase. All parents attended individual workshops due to difficulties scheduling group workshops, and service providers participated in either individual or group sessions. Workshops were conducted via Zoom with audio and video recording and lasted 2 h (with a 5–10 min break).

Workshops explored parent and service provider feedback on the preliminary Phase 1 design considerations to sense-check whether these reflected their needs and preferences. Feedback was then sought on the broad program design, including its four main components. Parents and service providers were then given access to the online component of the initial program prototype and encouraged to share their feedback aloud. Service providers were also provided with access to a prototype of the online component of the clinician training in this way. Follow-up questions explored participant preferences (e.g., what they liked, what they would change, and how design features should be structured and delivered) and how well the program addressed the design considerations.

#### 4.1.3. Data Analysis

Interviews were transcribed automatically via Zoom or by a third-party transcription service and checked by the first author for accuracy. Transcribed data were coded with the support of NVivo software, using Braun and Clarke’s six-phase reflexive thematic-analysis approach [64,65]. Data were analysed deductively using the five design considerations from Phase 1, as the purpose of this phase was to sense-check these themes and validate if and how the initial prototype met these needs. Additional themes were identified inductively to capture novel insights beyond these pre-defined themes. To avoid confirmation bias and ensure any novel insights would be captured, each transcript was initially coded openly before being coded to the Phase 1 themes. Throughout the analysis process, the research team also actively sought findings that may contradict the Phase 1 themes or suggest that the prototype did not meet these design considerations.

### 4.2. Phase 2 Results

Participants frequently reiterated findings from Phase 1, confirming the relevance of these for the program. For this reason, a summary of how well the initial prototype met the design considerations is provided in Supplementary File S1, with illustrative quotes. A novel theme was also developed, highlighting the program’s role in facilitating communication between parents and clinicians, and considerations for how this could occur. The results below outline this new theme as well as suggestions for improvement according to each design consideration.

#### 4.2.1. Building Readiness for Parenting Support

Suggestions for the next program iteration included a quick-exit button and extra mental health resources within module pages. Participants also suggested more clearly specifying for parents the program’s purpose, better highlighting the benefits of parent and clinician reflection questions, and introducing the program in terms of its potential benefits to parental mental health.

#### 4.2.2. Emotional and Social Support

Suggestions for the next program iteration included more explicitly acknowledging that parenting is difficult, additional 24/7 support options, more lived-experience examples, and additional prompts or opportunities to reach out to support networks or peers. Clinicians suggested that training should include ongoing group supervision and specify that clinician reflections are designed to be shared with other clinicians.

#### 4.2.3. Practical and Personalised Knowledge

Suggestions for the next program iteration included pictures of real people, space for parents to note personalised goals and strategies and answer goal prompts, and more example strategies within modules. Participants also recommended more goal options, a search function, and a personalised dashboard picture. Other suggestions included more contextualising of content to parenting with mental health challenges, more examples for fathers, and ensuring images include a balanced representation of different cultures.

#### 4.2.4. Parent-Led Empowerment

Suggestions for the next program iteration included space for parents to note extra comments or record their existing skills, while also ensuring parents have control over whether they share goals and reflections with clinicians. Participants recommended increasing and bolding encouraging language, removing language that may be deficit- or pressure-focused, and providing more obvious progress indicators.

#### 4.2.5. Accessible and Integrated Support

Suggestions for the next program iteration included improving the readability of content; minimising open text responses; more audio, including the option for text to be read aloud; versions in other languages; and encouraging parents to download content. They also suggested including videos and more images, colours, and activities. Parents recommended providing estimates of how long activities might take and acknowledging financial concerns as a potential challenge. Clinical leads recommended that parents be given a timeframe for program completion. Clinicians asked for a supervisor with parenting expertise and the option to contact parents to check in on progress between sessions.

#### 4.2.6. Emergent Theme: Bridge for Communication

Participants described how the program was able to facilitate communication between parents and clinicians. The end-of-module reflection questions were seen as helpful for starting and guiding sessions by communicating parent needs to clinicians. This feature was seen as an efficient way for parents to express priorities or sensitive issues that might be difficult to say aloud, though it was noted that these should not replace therapeutic conversation. Participants described the benefit of parents completing reflections while they were in the mindset of the program to help them record points to discuss later. Parents and clinicians appreciated clinicians having direct dashboard access to parent progress and reflections to facilitate easy communication, and clinicians’ ability to review these in advance for session preparation.

“Some people still find it hard to talk face-to-face… so I feel like this is really good because you’re getting this information to them. It’s like, when you fill out a questionnaire, and you almost feel anonymous… cause you’re not saying it. It’s another way of communicating without having to speak.”

[Parent 7]

Suggestions for the next iteration of the program included having more frequent reminders within the content to speak with clinicians and extra space for parents to note comments or questions to share. Clinical leads raised issues with clinicians having dashboard access to parent progress and reflections, including concerns about confidentiality, data security if a clinician or parent leaves the service, and added administrative burden. They concluded that parents should instead share their progress and reflections with clinicians directly unless logistical issues with the clinician dashboard can be resolved.

#### 4.2.7. Intervention Refinements

The research team refined the design of the initial prototype based on the Phase 2 findings. For example, sharing each module’s goals and reflection responses was made optional to give parents control over if and when they share this information with their clinician or other support people. Supplementary Table S5 provides a full outline of all prototype refinements as well as additional considerations for future iterations of the program beyond the scope of the current study.

As only a limited number of modules could be developed for this first iteration, topics were prioritised based on Phase 1 and 2 findings, our systematic review comparing parental factors between parents with and without anxiety and/or depression [48], and a broader literature review of parenting domains linked to parental mental health and child outcomes [5,45,67,68,69]. Particularly salient parenting domains identified from this search included talking with children about parental mental health, seeking help, self-care, parenting stress, effective discipline, aversiveness, withdrawal, warmth, parental self-efficacy, and parent–child relationship quality. The topics ‘Talking with children about parental mental health’ and ‘Showing affection and acceptance’ were chosen, with information on self-care and help-seeking integrated throughout these modules. These topics were selected to cover as many of the key domains as possible, while including content specific to parenting in the context of parental mental health challenges and prioritising warmth as a factor repeatedly linked to parent–child relationship quality and child outcomes [45,68].

Evidence-based content for the ‘Showing affection and acceptance’ module was retained from the original PiP Kids program, with features and wording adapted for contextualisation to parenting with mental health challenges. Content for the ‘Talking with children about parental mental health’ topic was divided into two modules, (1) ‘Getting ready for conversations’ and (2) ‘Having conversations‘, to keep each module manageable for parents. This new content was developed by reviewing existing resources on the topic [69,70,71,72,73,74,75,76]. The content and its presentation were comprehensively reviewed by research team members with expertise in psychology, parenting, and parental mental health. Modules were initially designed to take 10–15 min to complete; however, incorporating all design considerations while including evidence-based content required extending the duration of modules to approximately 15–25 min. Supplementary File S2 provides images of the refined parenting program prototype, including the dashboard and selected module pages.

## 5. Phase 3 Methods and Results: Deliver the Refined Prototype

### 5.1. Phase 3 Methods

#### 5.1.1. Participants

Recruitment followed the same methods as Phase 1, and parents from earlier phases were also invited to participate. Six parents expressed interest, including four from Phases 1 and 2. Five parents consented, including one newly recruited parent and the four returning participants. One of the returning participants withdrew due to competing life circumstances, and another was lost to follow-up after partially completing the prototype.

In total, three parents completed all three prototype modules and the interview, and their data were included for analysis. The parents were mothers (*n* = 2) and a grandmother (*n* = 1) aged 41–56 years (M = 47). The newly recruited parent was seeking mental health support at the collaborating health service, while the other two parents had lived experience of accessing support in the broader community within the past six months. Parents’ self-reported mental health challenges included high stress (*n* = 3), anxiety (*n* = 2), trauma (*n* = 2), and depression (*n* = 1). Parents’ reported cultural backgrounds included Australian/New Zealand (*n* = 2), East Asian (*n* = 1), and Middle Eastern (*n* = 1). Their highest education levels were one each of technical qualifications, a bachelor’s degree, and a postgraduate degree. All three were single parents and concession card holders. Supplementary Table S6 presents further demographic characteristics of the Phase 3 parent participants.

#### 5.1.2. Data Collection

Phase 3 focused on piloting the refined prototype with parents to explore its initial acceptability and identify areas for improvement. For this study, only the online module component of the refined prototype was piloted, not the clinician-support component or clinician training. A staged approach was considered valuable given the newly co-designed content and program features. This online-only trial served as a logical first step in evaluating the modules before integrating them with clinician support.

Parents completed a demographic questionnaire via REDCap before commencing the refined prototype. Parents were encouraged to complete one module per week, with the first author monitoring progress and sending weekly, personalised reminder emails based on each parent’s goals and progress. Approximately one month after commencing the prototype, parents completed a 1 hour semi-structured feedback interview. Interviews were conducted using a guide adapted from prior related research [47,77,78] to explore prototype acceptability and adaptations for the next iteration of the program. The interview schedule (Supplementary File S3) was adapted from the Theoretical Framework of Acceptability (TFA) questionnaire, a theory-informed approach to evaluating the acceptability of healthcare interventions [79,80]. Open questions asking parents for their perspectives on a clinician-support component were also included. Interviews were conducted one-on-one via Zoom with audio recording. Parents were reimbursed with a $35 gift card for the interview and an additional $10 for each online parenting module completed.

#### 5.1.3. Data Analysis

Interview recordings were automatically transcribed by Zoom and manually cleaned by the research team. Parents were invited to review their transcripts for alignment with their experience of the interview [81]; however, no parent chose to review their transcript. The transcripts were analysed with the support of NVivo using a reflexive thematic-analysis approach [64,65]. Themes were identified deductively using the seven constructs of the TFA [79,80], with subthemes developed inductively, including those not covered by the TFA but still relevant to acceptability.

### 5.2. Phase 3 Results

Themes reflecting the acceptability of the refined prototype were developed using the seven TFA constructs, with subthemes capturing specific contributors to acceptability and suggestions for improvement. Table 2 defines the TFA constructs and presents the subthemes with illustrative quotes.

#### 5.2.1. Affective Attitude

Parents expressed a positive experience with the program, including its self-directed, evidence-based content and optional further reading. They found it accessible and user-friendly with clear language, summaries, audio, and images to support understanding. The simple technology was appreciated, but parents recommended adding the ability to enlarge text and easily return to previous pages. Parents found the program engaging and interactive due to its varied text, visuals, and activities; however, they mentioned that interactive boxes could be more clearly indicated. The program was described as being supportive through non-judgmental and non-directive language, calming colours, and progress indicators, but parents suggested adding encouraging music when completing activities.

#### 5.2.2. Intervention Coherence

While parents generally had a good understanding of the program, they were occasionally uncertain about program instructions, such as whether some sections were informational or required a response. They suggested including a video to introduce the program, as written instructions may not be accessible for everyone. There was also some uncertainty about the program’s scope and users, as some parents expected extra modules and were unsure about who the program was for and how it would be accessed.

#### 5.2.3. Perceived Effectiveness

Parents reported that they felt the program helped strengthen their self-efficacy and self-belief by reinforcing their existing strengths. Parents said that reflective activities and practical suggestions prompted them to think about their parenting and make small adjustments. However, they stated that the end-of-module reflection questions did not encourage them to seek additional support as intended. Parents described the content as familiar, noting that while it included some new information, its main value was in reminding them of what they already knew. Parents suggested this may be most beneficial for new parents or those less familiar with the content. To further support reflection and practice, parents suggested including more topics, space for open reflection, and quizzes.

#### 5.2.4. Ethicality

Parents appreciated that the modules could be tailored to the needs of individual parents, such as being able to select strategies that were relevant and achievable for them. Parents also valued that the program was inclusive and represented diverse cultures in images, but they recommended depicting a broader range of family types and incorporating parenting examples from other cultures. They also mentioned that the content was not always catered to individual circumstances, such as low literacy, neurodivergence, or unique family contexts. Parents suggested ways to further tailor the program, such as uploading their own avatar and including space to note reflections specific to their situation.

#### 5.2.5. Burden

Parents had different experiences with the time and cognitive effort required for the program. They mentioned that it took around an hour to finish a module, which was longer than intended. Some found this manageable, but others found reading the text-heavy content demanding of their limited time and energy. Parents recommended shorter modules, less information displayed at once, faster page transitions, and more audio and video options. Although the self-directed modules allowed parents to fit the program flexibly around competing demands, they said that busy schedules still interrupted progress. Parents appreciated that all modules were available at once, and they found email reminders helpful, but suggested adding optional text message reminders.

#### 5.2.6. Opportunity Costs

All parents reported that participating in the program was worthwhile and required no sacrifices beyond time and internet access. However, one parent noted that internet access might be a challenge for some families.

#### 5.2.7. Self-Efficacy in Completing Program

Parents reported feeling confident they would complete the program, although uncertain how long it might take. Some open activities were perceived as difficult due to the need to put responses into words, and parents suggested more multiple-choice activities. Goals were seen as helpful and achievable, but applying them in daily life could be challenging given the need to problem-solve. Parents appreciated the goal reminders but suggested extra reminders and the ability to track goals on the dashboard.

#### 5.2.8. Additional Acceptability Subthemes

Parents valued that the program normalised parenting and mental health challenges and emphasised that no parent is perfect. They reinforced the importance of incorporating human support and connection, noting the benefit of flexible access to a clinician and peer-based social opportunities. One parent suggested incorporating opportunities for parents to receive feedback from their children on their parenting.

## 6. Discussion

This co-design study aimed to adapt an evidence-based digital parenting program (PiP Kids) for future implementation in an existing adult mental health service, to support parents seeking mental health care. Triangulation of findings across parents, clinicians, and clinical leads provided a multifaceted understanding of end-user needs, strengthened by the lived experience of several clinicians. The main findings identified from Phase 1 and reinforced in Phase 2 underpinned design considerations regarding the needs and preferences of parents and service providers. These design considerations are discussed in detail below, with reference to the existing literature and future research directions. Initial acceptability findings and suggestions for improvement from Phase 3 are integrated into the themes where relevant.

### 6.1. Building Parent and Clinician Readiness for Parenting Support

The first design consideration identified several factors impacting parent and clinician readiness for parenting support, including parent wellbeing and safety, clinician training, and both groups’ understanding of the purpose and value of the program. The Phase 1 and 2 findings regarding building parents’ readiness align with prior research emphasising that the immediate wellbeing of families should be addressed before they can engage in parenting support, particularly in contexts involving risk, safety concerns, or family violence [34]. Managing parental mental and physical health and engaging in self-care have also been identified as foundational for parenting capacity [17,82]. Participants from Phase 2 appreciated that the prototype helped support parent and family wellbeing through content that encouraged self-care and help-seeking, in addition to clinicians directly supporting parent mental health. Consistent with prior research, participants from Phase 1 viewed mental health treatment as the main role of the adult mental health service, with clinicians extending to parenting when it helped support the parent’s mental health goals or after acute concerns were addressed [24,29]. This suggests that parenting programs integrated within adult mental health services should prioritise parental mental health first, either by allowing treatment before the program begins or by supporting the parent’s mental health goals directly. Our adapted version of PiP Kids aims to address this by utilising a flexible, largely self-directed format that can be introduced at any stage of treatment, allowing clinicians to prioritise parental mental health goals while integrating parenting support as appropriate.

Additionally, our results suggest that a thorough introduction to the program may be particularly valuable for parents with mental health challenges, as this was expressed across all three phases. In the intended program design, this is supported through an introductory email sent to parents as well as clinicians introducing the program’s purpose, its intended benefits, and how to access and use it. We found that parents in Phase 2 particularly appreciated the clinicians’ role in supporting this introduction, similar to prior research [34]. However, it was repeatedly suggested that an initial video may also be beneficial. The importance of a clear introduction has also been found in previous research, with suggestions that setting clear expectations and describing the benefits of programs can help ease parent anxiety, support engagement, create stability, and minimise the uncertainty that has occurred across this and other programs for parents with mental health challenges [23,34].

Findings regarding clinician readiness also suggested the importance of clinicians understanding the program and feeling confident about its value and use. Clinicians from Phase 2 appreciated that the program would provide them with not just program training but also brief refreshers on parenting content and how their existing skills translate to parenting support. Since practitioner confidence and skills related to parenting are key factors in the likelihood of family-focused practice [83], this approach may support successful implementation into a range of settings, since it does not require extensive parenting-specific training [29]. Together, these findings suggest that integration of parenting support into adult mental health care should begin with a period of building readiness and addressing foundational factors with both parents and clinicians.

### 6.2. Facilitating Emotional, Social, and Peer Support for Parents and Clinicians

The second design consideration suggested that for parents, normalising non-judgmental emotional support and social connection is crucial, whereas for clinicians, professional peer support is required. In particular, initial acceptability feedback from parents in Phase 3 indicated that they found the supportive, non-judgmental language of the online modules helpful. Consistent with existing literature, parenting was described as a potentially sensitive topic, especially for parents with mental health challenges who often fear being judged as incompetent, which can act as a barrier to help-seeking [82,84]. This highlighted the importance of validating module content and empathetic emotional support from clinicians [85]. Specifically, across Phases 1 and 2, participants emphasised that parenting-related emotional support provided in an existing parent–clinician relationship would be particularly beneficial. This aligns with research suggesting the importance of establishing a trusting relationship prior to introducing parenting programs, to create a safe, non-critical, reflective environment for parents [34].

Across Phases 2 and 3, parents also valued that the module content encouraged social support from family and friends beyond the therapeutic alliance. This finding aligns with prior studies suggesting that parents benefit from social connection as it provides emotional support, shared parenting strategies, and support with practical parenting tasks [17,86]. Participants across all phases consistently reiterated the potential benefits of adding peer support from other parents with mental health challenges, which has been described by parents and clinicians previously [21,34,50]. Likely benefits include reduced social isolation as well as normalisation, reassurance, and learning from others in similar situations [23,84]. However, consistent with earlier findings [84,86], some participants in Phase 1 described the potential negative effects of peer groups, such as anxiety, shame, or reluctance to share personal information. It may be beneficial for future studies to investigate how to leverage existing technology to design peer support communities that enable the benefits of peer support while minimising the concerns articulated by participants.

Peer support was also valued by clinicians in Phases 1 and 2, as they highlighted the importance of connection with other clinicians delivering the program, similar to previous work [34]. Clinicians suggested that ongoing group supervision would be more valuable than a one-off workshop; however, clinical leads indicated this may be challenging in their busy health service. Future studies could explore implementation options for feasible group supervision, such as having a brief dedicated peer supervision or an embedded approach where parenting-related discussions are integrated within existing group supervision to reduce time demands on clinicians and services.

### 6.3. Providing Parents with Practical and Personalised Parenting Knowledge

The third design consideration highlighted the importance of providing parenting knowledge that is practical, relevant, and tailored to each parent’s unique family, context, and mental health challenges to help them implement strategies that work for them. This aligns with previous findings emphasising the benefit of practical strategies [82], clinician modelling [34], clinicians adapting content for each family [17,21], and support for problem-solving implementation of strategies [51,87]. Goals within the module content were also valued for helping parents action changes, consistent with research identifying goal setting as a key process in both parenting and mental health recovery [22]. During Phase 2, the written summaries of each counselling session were highlighted as a particularly helpful aspect for supporting implementation of learned strategies, but the usefulness of this in practice will need to be trialled in the future when piloting the clinician-support component. In Phase 3, parents suggested the helpfulness of the module’s practical tips, but noted that much of the content felt familiar, suggesting we may have reached parents with relatively high parenting literacy. This initial acceptability data suggested that for parents in this study, their main challenges were less about gaining parenting information, but more in relation to applying the content to their unique circumstances and putting strategies into action in their lives. Together, these findings suggest that even when module content is easy to understand, making changes in parenting may be the difficult part, especially for parents with mental health challenges [21]. This indicates a need for applied and personalised support beyond what a fully self-directed program can provide. Findings echo the potential benefit of clinician-support provided by the parent’s mental health clinician who already has the context of the parent’s family and mental health challenges for tailoring strategies [18].

Additionally, participants across Phases 2 and 3 liked the program’s consistent focus specifically on parenting in the context of mental health issues, which has been shown to be crucial to program engagement and acceptability for these parents [21,49]. For example, we and other studies have found the need for parenting programs to support parents in talking with their children about parental mental health [49,88], given children’s desire to receive this information from their parents and parents’ uncertainty about how to have these conversations [54,89]. These findings are consistent with past research, which suggests that generic parenting programs that fail to address parental mental health may be insufficient for parents with mental health concerns [21,43]. Finally, participants across all phases highlighted the importance of ensuring that module content reflects diverse family types and cultures, consistent with prior calls for inclusive and culturally sensitive design [29,51].

### 6.4. Encouraging Parent-Led Empowerment for Parents

The fourth design consideration demonstrated the importance of parents having autonomy and control while receiving the strengths-based support they need. Parents and service providers in Phase 2 described the value of the program’s self-directed module content for encouraging parent autonomy and independence, similar to prior work [34]; but they also reinforced the need for adjunct clinician support of such autonomy. Specifically, our initial acceptability data from Phase 3 indicated that fully self-directed programs may be less suitable for parents with mental health issues unless some degree of human support and encouragement is provided. These findings are consistent with emerging evidence suggesting that self-directed parenting programs may be acceptable for parents with mental health issues, but guidance is needed due to the potential impact of mental health symptoms on parents’ capacity to engage [34,51]. Incorporating clinician support to scaffold parents’ completion of an autonomous, self-directed program may not only enhance program engagement but also outcomes for parents and families [90]. Additionally, consistent with previous findings [29,82], we found the need for this clinician support to take a collaborative, parent-led approach to promote parent autonomy and empowerment. Parents have reported positive experiences when given the opportunity to participate in decisions about their care [91], reinforcing the importance of this finding.

Participants across all phases also emphasised the value of strengths-based support that acknowledges parents’ existing parenting strengths and fosters confidence in their parenting abilities. This aligns with previous evidence suggesting the importance of working with parents to promote a positive identity related to parenting [17,49]. To do this, findings echoed the need for interventions and clinicians to promote self-compassion and acceptance of imperfection, to help parents feel competent as parents and people [17,23]. Participants in Phases 2 and 3 appreciated the role of clinicians in promoting parent confidence and encouraging them to make progress with the program, consistent with prior work [34,82]. This focus on empowerment seems especially important given that feelings of inadequacy are common among parents with mental health challenges [86]. Initial acceptability data from Phase 3 suggested that parents felt the online modules supported their self-efficacy in parenting, similar to another study of a guided self-help parenting intervention [51]. Importantly, promoting parents’ self-efficacy, positive sense of self, agency, and independent problem-solving capacity have been identified as key influences on parenting changes [92] and mental health recovery [93], highlighting the value of interventions that target these aspects.

### 6.5. Embedding Accessible and Integrated Support into Existing Practices

The final design consideration highlighted that program delivery must be accessible and integrated into the everyday life of parents and clinicians. Across all phases, flexibility for parents to disengage and re-engage was valued to accommodate fluctuating symptoms and competing life demands, similar to prior suggestions for parents with mental health challenges [94]. Parents in Phases 2 and 3 found module features that reduced cognitive burden to be helpful, such as reminders, summaries, and multiple-choice questions. This is not surprising, given the barriers parents with mental health challenges face when engaging with parenting programs, including fluctuating mood, competing priorities, and memory difficulties [34]. Initial acceptability data from Phase 3 suggested that parents generally found the online modules to be clear, engaging, user-friendly, and interactive. However, our findings and those of others suggest that the accessibility of module content could be improved by minimising text, presenting content visually or in videos, and making the program available in other languages [34,40,51].

Parents in Phase 3 suggested making the modules shorter, similar to findings that brief interventions are particularly beneficial for parents facing social disadvantage [36]. Notably, attempts were made to break the ‘Talking with children’ module into two parts to make it more cognitively manageable; however, parents suggested that further breaking down modules into even smaller chunks would be ideal. Furthermore, parents stated that modules took them approximately one hour to complete, despite an intended design of 15–25 min, based on the original PiP Kids program. This discrepancy could suggest that parents with mental health challenges may require additional time for module completion due to factors such as difficulties with mental health symptoms, reading comprehension, engagement, or digital literacy. Parents from Phase 3 reported confidence in their ability to complete modules eventually; however, parents across all phases said that the largely self-directed program may not be appropriate for parents with low English proficiency, limited technology literacy, or reading difficulties, unless extra support is available to help with completing modules. Future iterations of the adapted program should explore ways to balance evidence-based PiP Kids content with parent preferences for brevity, such as by implementing suggestions including videos or bite-sized modules that were not feasible in the current project. To further guide the development of future iterations, we suggest prioritising core features to maintain program accessibility while minimising burden on parents. Features that are likely essential to retain, due to their reiteration across all study phases, include a clear program introduction, inclusivity of diverse family types and cultures, content specific to parenting in the context of parental mental health, strengths-based language, clinician and social support, flexibility to engage and re-engage, and features that reduce cognitive burden. The remaining features may offer added value, but whether they are essential remains to be tested in the future.

The need to embed the program into existing mental health practice was also emphasised across Phases 1 and 2, to ensure the program becomes a feasible and sustainable part of routine care. Our findings align with existing literature suggesting that parenting is closely intertwined with parent mental health recovery, providing challenging and positive experiences, hope, and motivation [91]. Parenting is a central factor in the lives of many parents, suggesting the need for parenting support to be integrated within individual mental health care, not isolated in a separate service [29,82]. This is supported by evidence that parents experience positive outcomes when parenting is included in mental health treatment [32,91] and that interventions that integrate parenting with mental health support may best serve parents with mental health issues [20]. Notably, parental mental health and parenting support are often delivered separately, with parents experiencing frustration when their parenting is not addressed by psychologists and they have to repeat themselves to multiple clinicians [91,95]. Our technology-assisted adaptation of PiP Kids aims to address this gap with the intention of embedding digital parenting support within existing mental health service provision. This approach has the potential to not only reduce the need for parents to engage with multiple providers but also allow clinicians to provide parenting support without delivering parenting content, unlike face-to-face parenting programs that often rely on clinician delivery [21,23]. A technology-assisted program such as this is thus likely to be beneficial in community mental health settings where clinicians often manage large caseloads, limited numbers of sessions, and competing demands [30,35].

Importantly, clinicians from Phase 2 indicated that the intended embedded program seems to align with existing service practices, including using existing clinician skills and clinicians referring parents to other services when support needs fall outside the scope. These referrals are likely to be especially important for parents with mental health issues who often present with complex needs, including child mental health problems and socioeconomic disadvantage [96,97]. Additionally, clinicians differed in their confidence levels regarding parenting, suggesting the need for clinician training that is flexible in not only timing but also content. Our self-directed clinician training with optional additional resources seemed to be well-regarded for addressing these needs once implemented. Overall, these findings suggest the need for there to be clear compatibility between parenting programs and service needs for successful integration of programs into routine practice.

### 6.6. Creating a Bridge for Communication Between Parents and Clinicians

A novel finding in Phase 2 of this study was the potential role of the program in facilitating communication between parents and clinicians. Previous research has shown that vignettes, written resources, or other program components can be useful for starting difficult parenting conversations [24,30]. Our findings extend this by suggesting that a technology-assisted parenting program has the potential to serve as a structured communication mechanism embedded within the service model, allowing parents to regularly articulate their parenting needs and experiences. However, preliminary acceptability findings from Phase 3 suggest that without integrated clinician support, the end-of-module reflection questions may not encourage parents to communicate their support needs to others. Future research might examine whether the program serves this communication function when clinician support is incorporated.

Moreover, the clinician dashboard, which would allow clinicians to view parent progress and end-of-module reflections, was seen by parents and clinicians in Phase 2 to have potential value for streamlining communication. However, clinical leads identified practical challenges for implementation, including confidentiality concerns and logistical issues when clinicians or parents leave the service. While identifying design considerations and assessing initial acceptability is a critical early step in this research, only these two clinical leads discussed the real-world feasibility of implementing the integrated program design. Thus, future work is needed to further explore how suggestions such as the clinician dashboard could be integrated and to identify facilitators and barriers to implementation of the program in everyday practice.

### 6.7. Limitations

This study has several limitations that should be considered when interpreting the findings. First, although parents had recent lived experience of seeking mental health care, several were not currently in treatment. This may have limited the study sample to parents with stable levels of mental health challenges, but not necessarily parents with current mental health challenges. Additionally, the recruited parents self-reported general mental health challenges such as anxiety and depression, and not all presentations seen at adult mental health services were represented (e.g., alcohol and other drug use, gambling, eating disorders). It may therefore be helpful for future evaluations to trial the program with parents who are all currently accessing mental health support and for a broader range of presentations.

Second, recruitment difficulties and dropout affected sample size, consistent with prior research noting problems engaging parents with mental health issues, potentially due to stigma or low energy [21,49]. Due to recruitment difficulties, we engaged parents from all over Australia, which may not reflect the demographics of the collaborating health service, potentially impacting the external validity of the findings. Third, all parent participants spoke English, were female, and were primarily mothers, as we were unable to recruit any fathers. All clinicians were also female, employed at a single organisation, and no clinicians from the alcohol and other drug (AOD) service could be recruited. This sample homogeneity further limits the generalisability of findings to other populations or service settings. Although the inclusion of only mothers is common in parenting research [51], it is important to repeat this research with fathers, linguistically diverse families, and clinicians from multiple service contexts to increase transferability. Additionally, although the participants had diverse cultural backgrounds, the socioeconomic background of participants was only assessed in Phase 3, and it is therefore unclear how socioeconomic diversity may have influenced Phase 1 and 2 findings.

Fourth, the collaborating health service already emphasises parenting within its model of care, consistent with the shift in Australia towards family-focused practice [98]. However, this may not be reflective of other services; for example, less than half of adult-mental health clinicians in a study in the United Kingdom worked on parenting with their parent clients [16]. Future research should explore whether findings differ in countries or services with less family-oriented care.

Fifth, for feasibility reasons, Phase 3 piloted only the online component of the adapted program. Although this precluded exploration of the implementation of the whole program, Phase 3 nonetheless provided valuable preliminary insights into areas for improvement in the online modules. Delivering the full intervention, including the clinician-support component, on a small-scale to assess implementation and clinical outcomes is thus a priority for the next phase of this research. Lastly, due to feasibility, only a limited number of content topics were included in this first iteration of the adapted program. Other important topics such as effective discipline, aversive parenting, and withdrawal should be a priority for inclusion in future iterations of the program.

## 7. Conclusions

To our knowledge, this is the first study to adapt a digital parenting program with the intention of embedding it within routine adult mental health care. It provides an in-depth exploration of the needs and preferences of parents seeking mental health support and the service providers who support them when it comes to technology-assisted parenting programs. The identified design considerations indicate the importance of readiness, support, personalisation, practical knowledge, empowerment, accessibility, and integration for designing helpful technology-assisted parenting programs for this population. Based on these findings, this study develops a unique model of care in which parents would access digital parenting content and adjunct clinician support from their existing mental health clinician, whilst minimising burden on clinicians. Such an approach advances the literature on family-focused practice by moving beyond siloed parent and child services to address the parent’s caregiving role within their mental health care, with the aim of better supporting parents and reducing the intergenerational risk of mental health issues. While the integrated, clinician-supported program is yet to be trialled, preliminary findings suggest the potential value of adopting this holistic, family-focused approach that integrates technology-assisted parenting support within adult mental health settings.

Although this study focused specifically on the PiP Kids program within one adult mental health service, the insights and design considerations may have broader implications for any technology-assisted parenting program aimed at supporting parents with mental health challenges. The results highlight the importance of co-designing these interventions with end users to ensure they are acceptable and accommodate parent and service needs. Collectively, findings contribute to implementation science in this field of research by exploring considerations for the future tailoring and implementation of digital parenting programs into real-world mental health services. Researchers, clinicians, and service developers seeking to design technology-assisted parenting programs for this population or implement interventions within existing mental health settings can consider our findings in their work.

## Figures and Tables

**Table 1:** Key components of the intended adapted parenting program and the clinician training package, developed through the translation of design considerations into program components.

Program Component	Description
Self-directed online modules	- Short 10–15 min, interactive online modules providing parents with evidence-based parenting knowledge and practical strategies in easy-to-understand, validating language.
End-of-module reflection questions	- Reflection questions at the end of each module to help parents set individualised goals, reflect on implementing goals, and identify areas where they may benefit from further support from clinicians or their broader support network.
Clinician-support component	- Clinician-support component integrated into parents’ existing mental health counselling sessions to supplement module content. Conversations are scaffolded by parents’ goals and reflection responses, which clinicians access via their own dashboard. Clinicians provide tailored guidance, support implementation of strategies, highlight parent strengths, and offer non-judgmental encouragement and emotional support.
Written summary of each counselling session	- A written summary of the key takeaways, goals, and plans from each counselling session, co-created between clinician and parent to help parents remember and implement learnings at home.
Clinician training package	- To support clinicians in delivering the clinician-support component. It includes (a) Self-paced online training modules introducing the program, the clinicians’ role, and each parenting topic (b) Access to the parent modules so they can explore the program content and delivery; (c) A one-off group workshop to discuss the training and practise skills with peers; (d) Ongoing supervision delivered by existing clinical supervisors within clinician’s regular supervision sessions.

**Table 2:** Acceptability themes, subthemes, and illustrative quotes for Phase 3.

TFA ^a^ Construct: Definition	Subtheme of TFA ^a^ Construct	Illustrative Quotes
Affective attitude: How an individual feels about the intervention.	Positive overall experience	- “All this online resources are self-guided evidence base. It’s really really good. And you always give the link to us where it’s from… so I think if people really interested, they… can go to do their own research.” [Parent 2]
	Accessible and user-friendly	- “There’s no difficulties… even though English is my second language, so… the language is really good. The content is easy to follow, easy to remember.” [Parent 2]
	Engaging and interactive	- “It was interactive, it was easy to interact with. It was different stuff… audio, text… big, small, different size… it just keeps you like, it’s not boring.” [Parent 3]
	Supportive and encouraging	- “Not in a way that I feel like they’re judging me they’re telling me what to do… It’s just some extra information… it was like easy to go ahead and friendly.” [Parent 3]
Intervention coherence: The extent to which the individual understands the intervention and how it works.	Some uncertainty about program instructions	- “Sometimes…English being my second language, I was not sure what I need to do when I read them, so it was not clear enough, like the instruction… Is it a question? Is this just information?… What do I need to do?” [Parent 3]
	Unclear program scope and users	- “Another thing was not clear to me is that at the end, who is going to use these resources… and is it self-referral, is it like advice by GP?” [Parent 3] - “I thought there were more [modules] coming.” [Parent 1]
Perceived effectiveness: The extent to which the intervention achieved its intended purpose.	Reinforced parent self-efficacy	- “It increased the knowledge of my strength… my belief in myself, and that, I do have the strength to do this… it just reaffirmed to me… That in the eyes of what people would recommend, I’m doing it.” [Parent 1]
	Encouraged some reflection and practice	- “It definitely give me a stop point to rethink what I have done, what I can improve, because there’s always room to improve. So not huge changes, but definitely helping to make small changes, adjustments.” [Parent 2]
	Content was familiar but helpful	- “I think it’s really really helpful for all parents across the board, no matter how much the parents already know the knowledge itself.” [Parent 2]
Ethicality: The extent to which the intervention has a good fit with an individual’s value system.	Ability to tailor to individual parents	- “I was able to choose my own goal, they didn’t choose it for me… If it was not reasonable or doable, I wouldn’t choose them.” [Parent 3]
	Inclusivity valued but could be improved	- “From the picture… it was inclusive… it was not just from one gender, one nationality it was kind of like inclusive.” [Parent 3] - “Four people family has been showing so many different pages, and I haven’t seen… families in other shapes… single parents, grandparents, same-sex parents, or just a traditional core mum and dad children.” [Parent 2]
Burden: The amount of effort required to participate in the intervention.	Time and cognitive effort required	- “It was so text based… it did take me a long time… I had to really concentrate… It’s not something that I could do while I had [child] around.” [Parent 1] - “It’s not too difficult. If I sit down for one hour, I can finish a module.” [Parent 2]
	Balancing competing life demands	- “I had set myself aside time to do it but life got in the way” [Parent 1] - “I’m working, I’m single mother I have a lot to do… sometimes those goals will be on top of my priority list sometimes there’s other things happening that they can’t wait so… to fit that in my busy schedule was difficult.” [Parent 3]
Opportunity costs: The benefits, profits, or values that were given up to engage in the intervention.	No sacrifices other than time and internet	- “Time, internet, some people they don’t have money to… like you need computer, internet and time.” [Parent 3] - “I didn’t have to sacrifice anything other than time.” [Parent 1]
Self-efficacy in completing program: The individual’s confidence that they can perform the behaviour(s) required to participate in the intervention.	Confidence in eventual completion	- “I was confident I’d finish it. But I wasn’t confident of when.” [Parent 1]
	Difficulty completing some program activities	- “The goal setting and the making it happen page, I didn’t really like them personally, because I hated school… I don’t like to have to write down all this stuff… and it’s like… How do I put that into words?” [Parent 1]
	Goals helpful but challenging to implement	- “I had to work out how to do it and it was just like, with the way [child]’s been lately. Concentrating on stuff… problem solving, and that sometimes there’s not, so clear for me how to do it.” [Parent 1] - “It was good that I have a goal… and it will benefit me… but on a negative side there was no manual … how to achieve this goal.” [Parent 3]
Additional acceptability subthemes:	Normalising challenges	- “The clear message is… No parents are perfect. You don’t have to be perfect. It’s okay to take a take a moment… we’re all humans” [Parent 2] - “It does normalise it because I wasn’t thinking mental health the whole time… I just viewed it as this is for parents.” [Parent 1]
	Desire for human support and connection	- "Ifwe have a live person behind this website… is it just got something for people to read?… Follow up outside this website would be very beneficial." [Parent 3] - “If there is sort of like an in-person opportunity, or possibly even a group… know that they can get together and talk about things.” [Parent 1]
	Opportunity for child feedback	- “Will that be a possible, children friendly versions like the little questionnaire or something?… So actually, I can get some feedback from my kids.” [Parent 2]

^a^ TFA = Theoretical Framework of Acceptability.

## Data Availability

The data from this study are not publicly available to protect participant privacy and anonymity. Deidentified summaries of the data are available on request from the corresponding author.

## Supplementary Materials

The following supporting information can be downloaded at: https://www.mdpi.com/article/10.3390/children13010129/s1, Table S1: Co-design methods and aims; Table S2: Demographics of Phase 1 parent and service provider participants; Table S3: Themes, subthemes and additional illustrative quotes for Phase 1; Table S4: Concrete features of the initial adapted parenting program alongside Phase 1 themes and subthemes; Table S5: Refinements made to the initial prototype and considerations for future iterations alongside Phase 2 themes; Table S6: Demographics of Phase 3 parent participants; File S1: Summary of Phase 2 findings regarding how well the initial prototype met the design considerations; File S2: Selected images of the refined parenting program prototype incorporating features from Phase 1 and 2 findings; File S3: Phase 3 parent interview schedule.

## Abbreviations

The following abbreviations are used in this manuscript:

PaRK	Parenting Resilient Kids
PiP	Partners in Parenting
PTSD	Post-Traumatic Stress Disorder
TFA	Theoretical Framework of Acceptability

## References

[1] Wolicki S.B., Bitsko R.H., Cree R.A., Danielson M.L., Ko J.Y., Warner L., Robinson L.R. (2021). Mental Health of Parents and Primary Caregivers by Sex and Associated Child Health Indicators. Adv. Res. Sci..

[2] Johnson S.E., Lawrence D., Perales F., Baxter J., Zubrick S.R. (2018). Prevalence of Mental Disorders Among Children and Adolescents of Parents with Self-Reported Mental Health Problems. Community Ment. Health J..

[3] Friedman H.P., Bilsky S.A., Luber M.J. (2023). Parent Anxiety, Child Anxiety, Parental Beliefs about Anxiety, and Parenting Behaviors: Examining Direct and Indirect Associations. J. Child Fam. Stud..

[4] Vreeland A., Gruhn M.A., Watson K.H., Bettis A.H., Compas B.E., Forehand R., Sullivan A.D. (2019). Parenting in Context: Associations of Parental Depression and Socioeconomic Factors with Parenting Behaviors. J. Child Fam. Stud..

[5] Christie H., Hamilton-Giachritsis C., Alves-Costa F., Tomlinson M., Halligan S.L. (2019). The Impact of Parental Posttraumatic Stress Disorder on Parenting: A Systematic Review. Eur. J. Psychotraumatol..

[6] Engur B. (2017). Parents with Psychosis: Impact on Parenting & Parent-Child Relationship- A Systematic Review. Glob. J. Addict. Rehabil. Med..

[7] Campbell L., Poon A.W.C., Ow R., Poon A.W.C. (2020). Parenting Challenges for Persons with a Serious Mental Illness. Mental Health and Social Work.

[8] Goodman S.H., Simon H.F.M., Shamblaw A.L., Kim C.Y. (2020). Parenting as a Mediator of Associations between Depression in Mothers and Children’s Functioning: A Systematic Review and Meta-Analysis. Clin. Child Fam. Psychol. Rev..

[9] Xu F., Cui W., Lawrence P.J. (2020). The Intergenerational Transmission of Anxiety in a Chinese Population: The Mediating Effect of Parental Control. J. Child Fam. Stud..

[10] Kluczniok D., Boedeker K., Hindi Attar C., Jaite C., Bierbaum A.-L., Fuehrer D., Paetz L., Dittrich K., Herpertz S.C., Brunner R. (2018). Emotional Availability in Mothers with Borderline Personality Disorder and Mothers with Remitted Major Depression Is Differently Associated with Psychopathology among School-Aged Children. J. Affect. Disord..

[11] Hine R.H., Maybery D.J., Goodyear M.J. (2018). Identity in Recovery for Mothers with a Mental Illness: A Literature Review. Psychiatr. Rehabil. J..

[12] Löchner J., Ulrich S.M., Lux U. (2024). The Impact of Parents’ Stress on Parents’ and Young Childrens’ Mental Health—Short- and Long-Term Effects of Risk and Resilience Factors in Families with Children Aged 0–3 in a Representative Sample. Stress Health.

[13] Phelan R.F., Howe D.J., Cashman E.L., Batchelor S.H. (2013). Enhancing Parenting Skills for Parents with Mental Illness: The Mental Health Positive Parenting Program. Med. J. Aust..

[14] Giannakopoulos G., Solantaus T., Tzavara C., Kolaitis G. (2021). Mental Health Promotion and Prevention Interventions in Families with Parental Depression: A Randomized Controlled Trial. J. Affect. Disord..

[15] Sandler I., Ingram A., Wolchik S., Tein J.-Y., Winslow E. (2015). Long-Term Effects of Parenting-Focused Preventive Interventions to Promote Resilience of Children and Adolescents. Child Dev. Perspect..

[16] Dunn A., Startup H., Cartwright-Hatton S. (2022). Adult Mental Health Service Engagement with Patients Who Are Parents: Evidence from 15 English Mental Health Trusts. Br. J. Clin. Psychol..

[17] Awram R., Hancock N., Honey A. (2017). Balancing Mothering and Mental Health Recovery: The Voices of Mothers Living with Mental Illness. Adv. Ment. Health.

[18] Solantaus T., Reupert A., Maybery D., Reupert A., Maybery D., Nicholson J., Göpfert M., Seeman M.V. (2015). Working with Parents Who Have a Psychiatric Disorder. Parental Psychiatric Disorder: Distressed Parents and Their Families.

[19] Everett Y., Martin C.G., Zalewski M. (2021). A Systematic Review Focusing on Psychotherapeutic Interventions That Impact Parental Psychopathology, Child Psychopathology and Parenting Behavior. Clin. Child Fam. Psychol. Rev..

[20] Barrett S., Muir C., Burns S., Adjei N., Forman J., Hackett S., Hirve R., Kaner E., Lynch R., Taylor-Robinson D. (2024). Interventions to Reduce Parental Substance Use, Domestic Violence and Mental Health Problems, and Their Impacts Upon Children’s Well-Being: A Systematic Review of Reviews and Evidence Mapping. Trauma Violence Abus..

[21] Isobel S., Meehan F., Pretty D. (2016). An Emotional Awareness Based Parenting Group for Parents with Mental Illness: A Mixed Methods Feasibility Study of Community Mental Health Nurse Facilitation. Arch. Psychiatr. Nurs..

[22] Maybery D., Reupert A., Goodyear M. (2015). Goal Setting in Recovery: Families Where a Parent Has a Mental Illness or a Dual Diagnosis. Child Fam. Soc. Work.

[23] Hestbaek E., Mikkelsen P.A., Thomas R.E., Sleed M., Holm C., Corlin A.B., Sørensen P., Væver M.S., Simonsen S. (2025). A Mentalization-Based Parenting Intervention (Lighthouse Parenting Programme) for Parents with Various Mental Disorders in Adult Mental Health Service: A Feasibility Study. Ment. Health Prev..

[24] McCarthy K.L., Lewis K.L., Bourke M.E., Grenyer B.F.S. (2016). A New Intervention for People with Borderline Personality Disorder Who Are Also Parents: A Pilot Study of Clinician Acceptability. Bord. Pers. Disord. Emot. Dysregul..

[25] Falkov A., Grant A., Hoadley B., Donaghy M., Weimand B.M. (2020). The Family Model: A Brief Intervention for Clinicians in Adult Mental Health Services Working with Parents Experiencing Mental Health Problems. Aust. N. Z. J. Psychiatry.

[26] Solantaus T., Toikka S. (2006). The Effective Family Programme: Preventative Services for the Children of Mentally Ill Parents in Finland. Int. J. Ment. Health Promot..

[27] Allchin B., Solantaus T. (2022). An Evidence-Based Practice Developed in-Situ: Let’s Talk About Children and a Consolidation of Its Evidence Base. Front. Psychiatry.

[28] Heyman M., Nicholson J., English K. (2024). The ParentingWell Practice Approach: Facilitating Implementation in U.S. Adult Mental Health Services. Front. Psychiatry.

[29] Nicholson J., Heyman M., English K., Biebel K. (2022). The ParentingWell Practice Approach: Adaptation of Let’s Talk About Children for Parents with Mental Illness in Adult Mental Health Services in the United States. Front. Psychiatry.

[30] Gray A.S., Townsend M.L., Bourke M.E., Grenyer B.F.S. (2019). Effectiveness of a Brief Parenting Intervention for People with Borderline Personality Disorder: A 12-Month Follow-up Study of Clinician Implementation in Practice. Adv. Ment. Health.

[31] Sherman M.D., Hooker S.A. (2018). Supporting Families Managing Parental Mental Illness: Challenges and Resources. Int. J. Psychiatry Med..

[32] Gregg L., Adderley H., Calam R., Wittkowski A. (2021). The Implementation of Family-focused Practice in Adult Mental Health Services: A Systematic Review Exploring the Influence of Practitioner and Workplace Factors. Int. J. Ment. Health Nurs..

[33] Riemersma I., Van Santvoort F., Van Doesum K., Hosman C., Janssens J., Van Der Zanden R., Otten R. (2022). ‘You Are Okay’: Effects of a Support and Educational Program for Children with Mild Intellectual Disability and Their Parents with Mental Health Concerns. J. Intellect. Disabil..

[34] Butler J., Gregg L., Calam R., Wittkowski A. (2021). Exploring Staff Implementation of a Self-Directed Parenting Intervention for Parents with Mental Health Difficulties. Community Ment. Health J..

[35] Ordway M.R., McMahon T.J., De Las Heras Kuhn L., Suchman N.E. (2018). Implementation of an Evidenced-Based Parenting Program in a Community Mental Health Setting. Infant Ment. Health J..

[36] Harris M., Andrews K., Gonzalez A., Prime H., Atkinson L. (2020). Technology-Assisted Parenting Interventions for Families Experiencing Social Disadvantage: A Meta-Analysis. Prev. Sci..

[37] Opie J.E., Esler T.B., Clancy E.M., Wright B., Painter F., Vuong A., Booth A.T., Newman L., Johns-Hayden A., Hameed M. (2024). Universal Digital Programs for Promoting Mental and Relational Health for Parents of Young Children: A Systematic Review and Meta-Analysis. Clin. Child Fam. Psychol. Rev..

[38] Hall C.M., Bierman K.L. (2015). Technology-Assisted Interventions for Parents of Young Children: Emerging Practices, Current Research, and Future Directions. Early Child. Res. Q..

[39] Breitenstein S.M., Gross D., Christophersen R. (2014). Digital Delivery Methods of Parenting Training Interventions: A Systematic Review: Digital Delivery of Parent Training. Worldviews Evid. Based Nurs..

[40] Ramos G., Chavira D.A. (2022). Use of Technology to Provide Mental Health Care for Racial and Ethnic Minorities: Evidence, Promise, and Challenges. Cogn. Behav. Pract..

[41] van der Zanden R.A., Speetjens P.A., Arntz K.S., Onrust S.A. (2010). Online Group Course for Parents with Mental Illness: Development and Pilot Study. J. Med. Internet Res..

[42] Jones S., Calam R., Sanders M., Diggle P.J., Dempsey R., Sadhnani V. (2014). A Pilot Web Based Positive Parenting Intervention to Help Bipolar Parents to Improve Perceived Parenting Skills and Child Outcomes. Behav. Cogn. Psychother..

[43] Kaplan K., Solomon P., Salzer M.S., Brusilovskiy E. (2014). Assessing an Internet-Based Parenting Intervention for Mothers with a Serious Mental Illness: A Randomized Controlled Trial. Psychiatr. Rehabil. J..

[44] Fernando L.M.N., Sim W.H., Jorm A.F., Rapee R., Lawrence K.A., Yap M.B.H. (2018). Parenting Resilient Kids (PaRK), an Online Parenting Program to Prevent Anxiety and Depression Problems in Primary School-Aged Children: Study Protocol for a Randomised Controlled Trial. Trials.

[45] Yap M.B.H., Jorm A.F. (2015). Parental Factors Associated with Childhood Anxiety, Depression, and Internalizing Problems: A Systematic Review and Meta-Analysis. J. Affect. Disord..

[46] Sim W.H., Fernando L.M.N., Jorm A.F., Rapee R.M., Lawrence K.A., Mackinnon A.J., Yap M.B.H. (2020). A Tailored Online Intervention to Improve Parenting Risk and Protective Factors for Child Anxiety and Depression: Medium-Term Findings from a Randomized Controlled Trial. J. Affect. Disord..

[47] Aldridge G., Wu L., Seguin J.P., Robinson J., Battaglia E., Olivier P., Yap M.B.H. (2024). Embedding Technology-Assisted Parenting Interventions in Real-World Settings to Empower Parents of Children with Adverse Childhood Experiences: Co-Design Study. JMIR Form. Res..

[48] Bennett M., McClure A., Khalesi H., Vu M.T.N., Feng J., Reupert A., Yap M.B.H. (2026). Differences in Parental Factors between Parents with and Without Depression or Anxiety Issues: A Systematic Review and Meta-Analysis. J. Affect. Disord..

[49] McFarland L., Fenton A. (2019). Unfogging the Future: Investigating a Strengths-Based Program to Build Capacity and Resilience in Parents with Mental Illness. Adv. Ment. Health.

[50] Coates D., Phelan R., Heap J., Howe D. (2017). “Being in a Group with Others Who Have Mental Illness Makes All the Difference”: The Views and Experiences of Parents Who Attended a Mental Health Parenting Program. Child. Youth Serv. Rev..

[51] Wolfenden L., Calam R., Drake R.J., Gregg L. (2022). The Triple P Positive Parenting Program for Parents with Psychosis: A Case Series with Qualitative Evaluation. Front. Psychiatry.

[52] Slattery P., Saeri A.K., Bragge P. (2020). Research Co-Design in Health: A Rapid Overview of Reviews. Health Res. Policy Syst..

[53] Morris H., O’Connor A., Cummins J., Valentine C., Dwyer A., Goodyear M., Skouteris H. (2019). A Pilot Efficacy Study of Parents Building Solutions: A Universal Parenting Program Using Co-Design and Strength-Based Approaches. Child. Youth Serv. Rev..

[54] Goodyear M., Zechmeister-Koss I., Bauer A., Christiansen H., Glatz-Grugger M., Paul J.L. (2022). Development of an Evidence-Informed and Codesigned Model of Support for Children of Parents with a Mental Illness—“It Takes a Village” Approach. Front. Psychiatry.

[55] Boddy C.R. (2016). Sample Size for Qualitative Research. Qual. Mark. Res. Int. J..

[56] Hawkins J., Madden K., Fletcher A., Midgley L., Grant A., Cox G., Moore L., Campbell R., Murphy S., Bonell C. (2017). Development of a Framework for the Co-Production and Prototyping of Public Health Interventions. BMC Public Health.

[57] Framework for Innovation—Design Council. https://www.designcouncil.org.uk/our-resources/framework-for-innovation/.

[58] Harris P.A., Taylor R., Thielke R., Payne J., Gonzalez N., Conde J.G. (2009). Research Electronic Data Capture (REDCap)—A Metadata-Driven Methodology and Workflow Process for Providing Translational Research Informatics Support. J. Biomed. Inform..

[59] Harris P.A., Taylor R., Minor B.L., Elliott V., Fernandez M., O’Neal L., McLeod L., Delacqua G., Delacqua F., Kirby J. (2019). The REDCap Consortium: Building an International Community of Software Platform Partners. J. Biomed. Inform..

[60] Yates S., Gatsou L. (2021). Idealisation and Stigmatisation of Parenting in Families with Parental Mental Illness. SSM Qual. Res. Health.

[61] Chan S.Y.Y., Ho G.W.K., Bressington D. (2019). Experiences of Self-stigmatization and Parenting in Chinese Mothers with Severe Mental Illness. Int. J. Ment. Health Nurs..

[62] Lister K., McFarlane R. (2021). Designing for Wellbeing: An Inclusive Learning Design Approach with Student Mental Health Vignettes. Open Prax..

[63] Törrönen J. (2018). Using Vignettes in Qualitative Interviews as Clues, Microcosms or Provokers. Qual. Res. J..

[64] Braun V., Clarke V. (2019). Reflecting on Reflexive Thematic Analysis. Qual. Res. Sport. Exerc. Health.

[65] Braun V., Clarke V. (2022). Thematic Analysis: A Practical Guide.

[66] Braun V., Clarke V. (2006). Using Thematic Analysis in Psychology. Qual. Res. Psychol..

[67] Daundasekara S.S., Beauchamp J.E., Hernandez D.C. (2021). Parenting Stress Mediates the Longitudinal Effect of Maternal Depression on Child Anxiety/Depressive Symptoms. J. Affect. Disord..

[68] Bernard K., Nissim G., Vaccaro S., Harris J.L., Lindhiem O. (2018). Association between Maternal Depression and Maternal Sensitivity from Birth to 12 Months: A Meta-Analysis. Attach. Hum. Dev..

[69] Grove C., Riebschleger J., Bosch A., Cavanaugh D., van der Ende P.C. (2017). Expert Views of Children’s Knowledge Needs Regarding Parental Mental Illness. Child. Youth Serv. Rev..

[70] In Focus: Talking with Children about Parental Mental Health Difficulties—Emerging Minds. https://emergingminds.com.au/resources/in-focus-talking-with-children-about-parental-mental-health-difficulties/.

[71] Talking to Your Primary School-Aged Child about Your Mental Health Difficulties—Emerging Minds. https://emergingminds.com.au/resources/talking-to-primary-school-aged-child-about-mh-difficulties/.

[72] Starting the Conversation About Your Mental Illness with Your Child—Emerging Minds. https://emergingminds.com.au/resources/starting-the-conversation-about-your-mental-illness-with-your-child/.

[73] Talking with Children About Their Parent’s Mental Health—Satellite Foundation. https://www.satellitefoundation.org.au/resources/talking-with-children-about-their-parents-mental-health/.

[74] Talking to Children of Primary School Age—COPMI. https://www.copmi.net.au/parents/helping-my-child-and-family/talking-about-mental-illness-with-your-child/talking-to-children-of-primary-school-age/.

[75] Riebschleger J., Grové C., Cavanaugh D., Costello S. (2017). Mental Health Literacy Content for Children of Parents with a Mental Illness: Thematic Analysis of a Literature Review. Brain Sci..

[76] Cooklin A. (2013). Promoting Children’s Resilience to Parental Mental Illness: Engaging the Child’s Thinking. Adv. Psychiatr. Treat..

[77] Smout A., Melvin G., Cardamone-Breen M., Jorm A., Xie J., Bartindale T., Olivier P., Seguin J., Wu L., Yap M.B.H. (2025). A Coach-Assisted, Online Parenting Programme to Support Parents of Adolescents Who Refuse School: Evidence of Acceptability and Feasibility. BJPsych Open.

[78] Cao A., Melvin G., Breen M.C., Salvaris C., Olivier P., Wu L., Seguin J., Xie J., Basur D., Thompson A. (2025). A Coach-Supported, Digital Parenting Program for Parents of Adolescents at Risk of Suicide: A Pilot Trial of Acceptability, Feasibility, Validity, and Short-Term Effects. BJPsych Open.

[79] Sekhon M., Cartwright M., Francis J.J. (2017). Acceptability of Healthcare Interventions: An Overview of Reviews and Development of a Theoretical Framework. BMC Health Serv. Res..

[80] Sekhon M., Cartwright M., Francis J.J. (2022). Development of a Theory-Informed Questionnaire to Assess the Acceptability of Healthcare Interventions. BMC Health Serv. Res..

[81] Motulsky S. (2021). Is Member Checking the Gold Standard of Quality in Qualitative Research?. Qual. Psychol..

[82] Cooper C., Tchernegovski P., Hine R. (2023). “The Validation Is Not Enough”: Australian Mothers’ Views and Perceptions of Mental Health Support from Psychologists in Private Practice. Clin. Psychol..

[83] Maybery D., Goodyear M., Reupert A.E., Grant A. (2016). Worker, Workplace or Families: What Influences Family Focused Practices in Adult Mental Health?. J. Psychiatr. Ment. Health Nurs..

[84] Radley J., Sivarajah N., Moltrecht B., Klampe M.-L., Hudson F., Delahay R., Barlow J., Johns L.C. (2022). A Scoping Review of Interventions Designed to Support Parents with Mental Illness That Would Be Appropriate for Parents with Psychosis. Front. Psychiatry.

[85] Overbeek M.M., Iozzia G., Maras A., Rijnberk C. (2022). Interventions for Parents with Mental Illnesses: A Scoping Review. Couple Fam. Psychol. Res. Pract..

[86] van der Ende P.C., van Busschbach J.T., Nicholson J., Korevaar E.L., van Weeghel J. (2016). Strategies for Parenting by Mothers and Fathers with a Mental Illness. J. Psychiatr. Ment. Health Nurs..

[87] Nicholson J., Albert K., Gershenson B., Williams V., Biebel K. (2009). Family Options for Parents with Mental Illnesses: A Developmental, Mixed Methods Pilot Study. Psychiatr. Rehabil. J..

[88] Afzelius M., Plantin L., Östman M. (2018). Families Living with Parental Mental Illness and Their Experiences of Family Interventions. J. Psychiatr. Ment. Health Nurs..

[89] Grove C., Reupert A., Maybery D. (2015). Gaining Knowledge about Parental Mental Illness: How Does It Empower Children?: ‘Family Focus’: A DVD Intervention. Child Fam. Soc. Work.

[90] Day J.J., Sanders M.R. (2018). Do Parents Benefit from Help When Completing a Self-Guided Parenting Program Online? A Randomized Controlled Trial Comparing Triple P Online with and Without Telephone Support. Behav. Ther..

[91] Jones M., Pietilä I., Joronen K., Simpson W., Gray S., Kaunonen M. (2016). Parents with Mental Illness—A Qualitative Study of Identities and Experiences with Support Services. Psychiatr. Ment. Health Nurs..

[92] Sanders M.R., Turner K.M.T., Metzler C.W. (2019). Applying Self-Regulation Principles in the Delivery of Parenting Interventions. Clin. Child. Fam. Psychol. Rev..

[93] Dell N.A., Long C., Mancini M.A. (2021). Models of Mental Health Recovery: An Overview of Systematic Reviews and Qualitative Meta-Syntheses. Psychiatr. Rehabil. J..

[94] Reupert A., Maybery D. (2011). Programmes for Parents with a Mental Illness: Programmes for Parents with a Mental Illness. J. Psychiatr. Ment. Health Nurs..

[95] Al Sager A., Goodman S.H., Jeong J., Bain P.A., Ahun M.N. (2024). Effects of Multi-Component Parenting and Parental Mental Health Interventions on Early Childhood Development and Parent Outcomes: A Systematic Review and Meta-Analysis. Lancet Child Adolesc. Health.

[96] Oliver-Parra A., Dalmau-Bueno A., Ruiz-Muñoz D., García-Altés A. (2020). Relationship between Parents’ Mental Disorders and Socioeconomic Status and Offspring’s Psychopathology: A Cross-Sectional Study. PLoS ONE.

[97] Pierce M., Abel K.M., Muwonge J., Wicks S., Nevriana A., Hope H., Dalman C., Kosidou K. (2020). Prevalence of Parental Mental Illness and Association with Socioeconomic Adversity among Children in Sweden between 2006 and 2016: A Population-Based Cohort Study. Lancet Public Health.

[98] Foster K., Maybery D., Reupert A., Gladstone B., Grant A., Ruud T., Falkov A., Kowalenko N. (2016). Family-Focused Practice in Mental Health Care: An Integrative Review. Child Youth Serv..

