# Supplementary material for: Using Co-Design to Adapt a Digital Parenting Program for Parents Seeking Mental Health Support
Source: Children (Basel). 2026 Jan 15;13(1):129. doi: 10.3390/children13010129 (PMC12840143; doi:10.3390/children13010129)
Supplement: Supplementary file 1 [file children-13-00129-s001.zip › children-4066491-supplementary.pdf]

Using co-design to adapt a digital parenting program for parents seeking mental health support

Supplementary Materials

Table of Contents

Supplementary Table S1. Co-design methods and aims....2

Supplementary Table S2. Demographics of Phase 1 parent and service provider participants....4

Supplementary Table S3. Themes, subthemes and additional illustrative quotes for Phase 1....5

Supplementary Table S4. Concrete features of the initial adapted parenting program alongside Phase 1 themes and subthemes....6

Supplementary File S1. Summary of Phase 2 findings regarding how well the initial prototype met the design considerations....9

Supplementary Table S5. Refinements made to the initial prototype and considerations for future iterations alongside Phase 2 themes....12

Supplementary File S2. Selected images of the refined parenting program prototype incorporating features from Phase 1 and 2 findings....15

Selected images from Module 1:....15

Selected images from Module 2:....18

Selected images from Module 3:....19

Supplementary Table S6. Demographics of Phase 3 parent participants....20

Supplementary File S3. Phase 3 parent interview schedule....21

Using co-design to adapt a digital parenting program for parents seeking mental health support

Supplementary Table S1. Co-design methods and aims

**Method**	**Aims**
**Phase 1 (*Discover* and *Define*):** Identify design considerations for integrating a technology-assisted parenting program into an existing mental health service by understanding the needs and preferences of parents and service providers.	
Parent co-design workshop	Discuss key parenting challenges faced by parents seeking mental health support and identify strategies for supporting parents with these challenges.<br>Understand barriers faced by parents in engaging with parenting programs and in applying parenting knowledge in daily life and identify strategies for supporting parents to overcome these barriers.
Clinician co-design workshop (Round 1)	Map current service provision and clinician roles within the collaborating mental health service to identify key enablers and constraints for integration of a technology-assisted parenting program.<br>Prioritise key challenges faced by parent clients in applying parenting knowledge in daily life and explore opportunities and barriers for addressing these challenges within the service.
Clinical Lead co-design workshop (Round 1)	Explore how a technology assisted parenting program could be integrated within existing service delivery, including desirable features and required organisational resources.<br>Prioritise key challenges faced by parent clients in applying parenting knowledge in daily life and explore opportunities and barriers for addressing these challenges within the service.
Clinician co-design workshop (Round 2)	Explore clinician feedback on emerging themes from prior parent, clinician and clinical lead workshops.<br>Understand the feasibility of clinicians undertaking parenting support tasks within their role and identify resources, training and support required to increase clinician confidence in completing these tasks.
Clinical Lead co-design workshop (Round 2)	Explore clinical lead feedback on emerging themes from prior parent, clinician and clinical lead workshops.<br>Understand organisational resources that are currently available or could be made available to support clinicians in completing parenting support tasks.

Method	Aims
**Phase 2 (*Develop*):** Create an initial prototype parenting program that addresses the design considerations and validate these themes and prototype with parents and service providers.	
Parent co-design workshop	Explore parent feedback on preliminary design consideration findings from all workshops to date.<br>Iteratively develop and present parents with an initial prototype parenting program based on the design considerations.<br>Understand if and how the initial prototype met parent needs and preferences for a technology-assisted parenting program and refine the prototype based on feedback.
Clinician co-design workshop	Explore clinician feedback on preliminary design consideration findings from all workshops to date.<br>Iteratively develop and present clinicians with an initial prototype parenting program and associated clinician training based on the design considerations.<br>Understand if and how the initial prototype met parent and service provider needs for a technology-assisted parenting program and refine the prototype based on feedback.
Clinical Lead co-design workshop	Explore clinical lead feedback on preliminary design consideration findings from all workshops to date.<br>Iteratively develop and present clinical leads with an initial prototype parenting program and associated clinician training based on the design considerations.<br>Understand if and how the initial prototype met parent and service provider needs for a technology-assisted parenting program and refine the prototype based on feedback.
**Phase 3 (*Deliver*):** Pilot the refined prototype parenting program with parents and collect feedback to explore its acceptability and identify further adaptations for the next iteration.	
Pilot prototype	Provide parent end-users with the online component of the refined prototype program on a small-scale.
Parent interview	Conduct qualitative interviews with parents to explore the acceptability of the program prototype and identify further adaptations for the next iteration of development.

Supplementary Table S2. Demographics of Phase 1 parent and service provider participants

Demographic characteristic	Parents (n = 8)	Clinicians (n = 5)	Clinical Leads (n = 2)
**Age in years, mean (SD; range)**	41.1 (2.90; 39-46)	37.8 (9.52; 25-50)	55.5 (4.95; 52-59)
**Gender, n (%)**			
Women	8 (100%)	5 (100%)	2 (100%)
**Ethnic/Racial background, n (%) ***			
Australian / New Zealand	4 (50%)	1 (20%)	1 (50%)
East Asian (e.g., China, Japan)	1 (12.5%)	0 (0%)	0 (0%)
European (e.g., United Kingdom, Macedonia)	2 (25%)	1 (20%)	1 (50%)
Middle Eastern (e.g., Turkey, Iran)	1 (12.5%)	1 (20%)	0 (0%)
Pacific Islander (e.g., Fiji)	2 (25%)	1 (20%)	0 (0%)
South Asian (e.g., India, Sri Lanka)	1 (12.5%)	1 (20%)	0 (0%)
Māori	0 (0%)	1 (20%)	0 (0%)
**Number of children, mean (SD; range)**	1.6 (0.52; 1-2)	-	-
**Child age in years, mean (SD; range)**	7.9 (2.90; 3-14)	-	-
**Parent self-reported mental health challenges, n (%) ***			
High stress	7 (87.5%)	-	-
Anxiety	5 (62.5%)	-	-
Depression	4 (50%)	-	-
Trauma or posttraumatic stress disorder	3 (37.5%)	-	-
Psychosis	1 (12.5%)	-	-
Grief	1 (12.5%)	-	-
Bipolar disorder	1 (12.5%)	-	-
**Time since last psychological support, n (%)**			
Currently accessing support (< 1 month)	3 (37.5%)	-	-
1 - 3 months ago	3 (37.5%)	-	-
4 - 6 months ago	1 (12.5%)	-	-
7 - 9 months ago	1 (12.5%)	-	-
**Parent state of residence, n (%)**			
Victoria	3 (37.5%)	-	-
South Australia	2 (25%)	-	-
Queensland	2 (25%)	-	-
New South Wales	1 (12.5%)	-	-
**Service provider professions, n (%)**			
Counsellor	-	2 (40%)	0 (0%)
Social Worker	-	3 (60%)	0 (0%)
Team Leader + Psychologist	-	0 (0%)	1 (50%)
Service Manager + Social Worker	-	0 (0%)	1 (50%)
**Primary area of practice, n (%)**			
Mild-to-severe mental health challenges	-	4 (80%)	1 (50%)
Gambling concerns	-	1 (20%)	0 (0%)
Service management	-	0 (0%)	1 (50%)
**Years of mental health experience, mean (SD; range)**	-	8 (7; 1-16)	27.5 (3.54; 25-30)

* *Note:* Participants could select more than one ethnic/racial background or mental health issue; these percentages do not sum to 100%.

Using co-design to adapt a digital parenting program for parents seeking mental health support

Supplementary Table S3. Themes, subthemes and additional illustrative quotes for Phase 1

Phase 1 Theme	Subtheme	Illustrative quotes
**Building readiness for parenting support**	Parental wellbeing foundations	- "Focusing on that self-care piece… we focus so much on trying to be a good parent and not being a failure, but we often are better at parenting when we have focused on ourselves." [Parent 6]
	Program understanding and confidence	- "If she had a snippet of what could be achieved or how [the program] could be helpful or what she might resonate with... she might prioritise it." [Parent 5]<br>- "All practitioners… have the skills… they do have an understanding of child development… around relationships. They have all that. So it's just using this tool also to just feel like they're… developing their confidence as well, to be able to integrate that." [Clinical Lead 2]
**Emotional and social support**	Emotional support and validation	- "It would help her to feel reassured that her feelings are probably normal. That family life is hard. That… emotions mean something." [Parent 7]<br>- "The foundation of addressing all of these is… not to make the parent feel judged. I feel once you have that engagement and rapport and the kindness and respect in the relationship you know that active listening." [Clinician 1]
	Social and peer connection	- "A big thing is like the connection, just checking in around that kind of broader system, again around who else is… able to help you with the children… so it's not all on them." [Parent 6]<br>- "Sometimes this group, they have more negative effect rather than positive effect" [Parent 5]
**Practical and personalised knowledge**	Practical knowledge	- "Being able to practice it, cause you have to be able to do it… learning is one thing, but practical is another. [Parent 3]<br>- "Building a bit of an action plan… some goal that they would like to have achieved… a personal plan." [Parent 6]
	Personalised knowledge	- "It's important to help the individual understand that this isn't just generic, this is for you. Like this is something that you are going through… and this will help you right now, rather than just a generic, oh this is a great parenting course." [Parent 7]<br>- "It depends on the parent and where they're at, their level of… mental health… It's really gauging as well based on the parents needs and what's happening for that parent as to whether that particular recommendation would be appropriate at that time." [Clinician 1]
**Parent-led empowerment**	Guided autonomy	- "We can feed them the information, but I think it's much more powerful… giving them that time to reflect... So they will come up with the solutions themselves." [Parent 6]<br>- "I'm also conscious of who my client is and what they want to focus their sessions on… if they really want to… reflect on themselves and their emotions, I don't want to push them to… focus on your children." [Clinician 2]
	Parent self-efficacy and self-worth	- "If she's feeling… disempowered by her experience then what is a way that she can gain some of that back… having a bit of a plan with… achievable tasks and actions, like really basic stuff just to bring her back down to sort of practical day-to-day stuff. And that might fill her just with a little bit more confidence" [Parent 5]<br>- "Reassure her and advise her that she's doing the best she can in the situation… there's no… perfect way of parenting. Everyone does their best." [Parent 8]
**Accessible and integrated support**	Low-burden accessibility	- "These commitments are difficult, for busy parents, so if they have online courses under her own time that would be very helpful… So flexible with time is very helpful… and also hopefully there's not much cost involved, cause that can be a barrier... And some people got language barrier as well." [Parent 4]<br>- "Designed in a way that is broken up in different styles… Beautiful kind of visual pieces and then mixed in with… recorded transcript or you can just read it… Something that is visually appealing sometimes and other times just the straight up black and white stuff to keep you focused." [Parent 5]
	Seamless integration	- "They've got a tool that they can do at home when they don't see the clinician. Cause usually we see clients fortnightly, we don't see them weekly, so they've got a tool that they can have at home they can work on as well." [Clinical Lead 2]<br>- "If it's something that requires a bit more skill than what I have, I would definitely be referring." [Clinician 5]

Supplementary Table S4. Concrete features of the initial adapted parenting program alongside Phase 1 themes and subthemes

Phase 1 Theme & Subtheme	Concretised features for the initial parenting program prototype to address each finding*
**Building readiness for parenting support**	
Parental wellbeing foundations	• Overall program design: largely self-directed program allows clinician to continue focusing primarily on parent wellbeing during mental health sessions.<br>• Module content: 'Useful Resources' page including links to crisis and mental health resources to support parent and family wellbeing.<br>• Module content: sporadically encourages parents to seek help for their mental health, take time out from parenting and engage in self-care for physical and mental health.<br>• Clinician-support: clinician to provide parents with mental health support and assess and manage risk as per their role as mental health clinician.
Program understanding and confidence	• Clinician-support: clinician to introduce parents to the program including providing information about its purpose, benefits, how to access and use it etc.<br>• Clinician training: provides clinicians with an introduction to the program structure, purpose, benefits, evidence base, how to use it and the clinician's role in the program.<br>• Clinician training: provides clinicians with relevant information on each parenting topic including rationale, developmentally appropriate examples, common parenting challenges and strategies for supporting parents.<br>• Clinician training: provides clinicians with access to the online modules that parents complete so they can effectively guide parents through completion of the program.<br>• Clinician training: highlights clinicians existing skills that are transferable to parenting, to build clinician confidence.
**Feeling understood, supported and connected**	
Emotional support and validation	• Overall program design: clinician support component integrated within counselling sessions with clinicians who have an existing therapeutic relationship with parents.<br>• Module content: normalising, validating and emotionally supportive language used.<br>• Module content: 'Useful Resources' page including links to helplines to provide parents with emotional support outside of working hours.<br>• Clinician-support: clinician to provide non-judgmental emotional support through counselling sessions including validation and normalisation.
Social and peer connection	• Module content: lived experience case vignettes of parents with experience of parenting with mental health challenges.<br>• Module content: parents are sporadically encouraged to reach out to their support networks (family, friends, professionals) for connection and practical help.<br>• Clinician training: includes a one-off group workshop where clinicians can connect with peers through discussing training modules and practicing supporting parents.<br>• Clinician training: includes reflection questions for clinicians to complete and share with their peers during the group workshop.
**Contextualised and applied knowledge**	
Practical knowledge	• Overall program design: parents to take home a written summary of the key takeaways, plans and goals discussed in counselling sessions to support implementation at home.<br>• Module content: provides practical examples/tips for implementing parenting strategies.

Phase 1 Theme & Subtheme	Concretised features for the initial parenting program prototype to address each finding*
	• Module content: activities within each module encouraging parents to practice strategies with their kids or roleplay with others and engage in goal setting and planning.<br>• Module content: automated email reminders sent by the program each week prompt parents to work towards implementing their goals.<br>• Clinician-support: clinician to roleplay or practice strategies with parents during sessions, assist with problem solving barriers, answer questions, and help set goals and develop practical steps for implementing them.
Personalised knowledge	• Overall program design: Parents complete reflection questions at the end of each module to set goals and identify areas where they may need extra support to achieve these goals. Clinicians can access these responses via their own dashboard to tailor sessions to each parent's specific support needs.<br>• Module content: options provided within each strategy and goal to allow parents to select the best approach for them and their family.<br>• Module content: acknowledges that every family, parent and child is different.<br>• Module content: content consistently contextualised to parenting while experiencing mental health challenges, including example topics specifically relevant to parental mental health (e.g., talking to children about parent mental health, self-care, seeking help).<br>• Module content: content and strategies are designed to be culturally inclusive/adaptable to ensure relevance for diverse backgrounds.<br>• Clinician-support: clinician to use their understanding of the parent client's life, mental health and family circumstances to recommend helpful modules and tailor strategies.
**Parent-led empowerment**	
Guided autonomy	• Overall program design: Parents complete reflection questions at the end of each module to set goals and identify areas where they want extra support. Clinicians to review responses on their own dashboard to ensure sessions are guided by parent priorities.<br>• Module content: activities integrated within modules encourage parents to independently reflect and brainstorm own solutions.<br>• Module content: all modules are unlocked, and every page is optional, so parents choose the modules they want to complete and the order they complete them.<br>• Clinician-support: clinician to scaffold parents to use and build on their existing knowledge and generate their own solutions.<br>• Clinician-support: flexibility to spend as little or as much time of the parent's regular counselling sessions discussing the parenting program, depending on parent preferences.
Encouraging self-efficacy and self-worth	• Module content: strengths-based, encouraging language incorporated throughout which encourages imperfect or 'good enough' parenting.<br>• Module content: provides strategies and examples that are achievable for parents struggling in their mental health e.g., goal setting focuses on one small task at a time.<br>• Module content: every module has a final page dedicated to encouraging self-compassion and patience with progress, accepting parenting mistakes and reducing self-blame.<br>• Module content: each module contains a progress bar on the dashboard as well as a sidebar within the module that ticks off pages as parents complete them, helping parents to monitor their progress.<br>• Clinician-support: clinician to offer parents encouragement and help them acknowledge and build on their strengths, notice their progress and celebrate small wins.

Phase 1 Theme & Subtheme	Concretised features for the initial parenting program prototype to address each finding*
**Accessible and integrated delivery**	
Low burden accessibility	• Overall program design: short, 10-15-minute self-directed online modules that can be accessed across multiple devices (e.g., phone, tablet, computer), stopped and started, completed at any time and at the parent's own pace.<br>• Overall program design: program utilises a user-friendly interface including a simple dashboard, easy access to modules, intuitive navigation and low burden activities (e.g., true/false quiz and multiple-choice reflection questions).<br>• Module content: no financial cost for participating in the program and many of the program's suggested strategies require low or no financial cost to implement.<br>• Module content: written in easy-to-understand language and divided across module pages with text, interactive activities, visuals, audio and quizzes to create engaging, easy to follow modules.<br>• Module content: automated email reminders sent by the program each week help parents maintain engagement by keeping the program front of mind despite other priorities.<br>• Module content: encouraging parents to download a copy of their responses to reflection questions at the end of each module to make it easy to re-visit and remember their goals and areas where they would like to seek additional support.
Seamless integration	• Overall program design: clinician support component integrated within existing counselling sessions to minimise out of session commitment from clinicians.<br>• Overall program design: largely self-directed program minimises clinician burden, saves time in sessions by allowing parents to complete learning and reflection beforehand, and ensures clinicians can focus on applying their existing clinical skills rather than delivering parenting content.<br>• Module content: 'Useful Resources' page to provide a list of extra services that parents can access if they need additional support (e.g., for child mental health, family separation, domestic violence, homelessness etc.).<br>• Clinician-support: clinician to use secondary consultation and refer parents to additional professional support or more intensive parenting support as needed.<br>• Clinician training: delivered primarily via brief self-directed online modules with links to additional optional resources to provide flexibility to complete on any day, at their own pace, revisit content and access more information as needed.<br>• Clinician training: ongoing supervision delivered by existing clinical supervisors within clinician's regular supervision sessions, provided the supervisor has completed program training and has experience supporting parents with their parenting).

* Each feature has been classified into one of four categories to describe where it was enacted within the parenting program design: 1) overall program design = features comprising the structure, format and delivery of the program as a whole; 2) module content = features incorporated within the online module component; 3) clinician-support = features to be enacted by the clinician support component; and 4) clinician training = resources and support to be provided to clinicians to help them support parents in engaging with the parenting program.

Using co-design to adapt a digital parenting program for parents seeking mental health support

Supplementary File S1. Summary of Phase 2 findings regarding how well the initial prototype met the design considerations

The summary below provides a description of the Phase 2 findings regarding how well the initial prototype met each of the design considerations. Illustrative quotes are provided in the table.

Building readiness for parenting support

Parents and service providers appreciated that the program supports parental wellbeing, by encouraging self-care and help-seeking. Participants valued the clinician's role in providing parents with mental health support and they liked that parent reflection questions would help clinicians assess risk and mental health needs.

All participants valued the role of clinicians in introducing the program to parents and appreciated that the content acknowledges that starting is challenging. Clinicians valued training on not just the program but also parenting content. They particularly liked that training highlights how their skills translate to parenting and includes reflection questions to gauge their confidence and areas for improvement.

Feeling understood, supported and connected

Participants valued the program's emotionally supportive approach including its validating language and normalisation of mental health and parenting challenges. They liked the human touch of clinician support and the 24/7 helplines given the sensitive nature of parenting. Clinicians providing non-judgmental support within an existing therapeutic relationship was also highly regarded.

Parents and service providers appreciated that the program fosters parent social support through audio examples of parents with lived experience and encouraging parents to reach out to existing support networks. Clinicians valued that peer support had been incorporated into their training and that reflection questions provided opportunities to learn from peers.

Contextualised and applied knowledge

Participants valued the program's practical and tailored design, with existing clinicians helping break strategies into steps, problem solve barriers and adapt content to parents' family, cultural, and mental health needs. They appreciated that parents are encouraged to set personalised goals and practise strategies, with end-of-module reflection questions helping to identify individual support needs. Practical tools such as quizzes and concrete examples were highly regarded for consolidating learning. Written session summaries were valued for reinforcing goals and strategies.

Participants appreciated the program's personalisation including clinician support in recommending modules and multiple options to accommodate diverse

parenting styles. They liked that content acknowledges each family is different and is specific to parents with mental health concerns, while remaining broad enough for all families. Inclusive images and the ability for any family member to use the program were valued for reflecting diverse families.

Parent-led empowerment

Participants appreciated that the program balances parent autonomy with clinician guidance and includes activities for independent problem solving. They liked that the program is parent-led as parents can choose which optional activities or resources to engage with and whether to discuss parenting in sessions. Participants appreciated that clinicians would coach rather than dictate and would encourage parents to generate their own solutions.

Parents and service providers liked that the program promotes parent self-efficacy and self-worth through strengths-based, encouraging language, and achievable strategies. They appreciated the progress indicators, encouragement of imperfect parenting and self-compassionate language for reducing pressure and self-blame. Clinicians providing encouragement and acknowledging existing strengths and achievements was also valued. Written session summaries were appreciated for reinforcing accomplishments and taking home clinician encouragement.

Accessible and integrated delivery

Participants highlighted the program's accessible design, including short modules and flexibility to stop and start at any time. They valued features that reduce cognitive load, such as pre-listed options, downloadable goals, session summaries and reminders. Participants liked the easy-to-read content including simple language and short paragraphs with bolding or colours. They appreciated the low-cost examples of parent-child activities, user-friendly technology, audio and interactive elements. Participants emphasised that competing priorities may still restrict program use and that parents with limited English proficiency or low literacy may need additional support or a different program.

All participants appreciated the program's seamless integration in the mental health services including parenting check-in incorporated into existing sessions. They valued the program as a time efficient tool which allows parents to progress without clinicians. Participants appreciated the program's alignment with existing practices including setting homework, using clinicians' existing skills and providing referrals when needed. Clinician training was also seen as well-integrated due to its self-directed structure, flexible templates, guidance on balancing parenting and mental health support, and simple technology. Clinical leads liked having program supervision integrated into existing supervision and clinicians noted this as acceptable if supervisors were trained in the program.

Phase 2 Theme	Subtheme		Illustrative quotes
**Building readiness for parenting support**	Parental wellbeing foundations	-	"I like the wording around considering it a strength to recognise when you need to take time and space for yourself… As parents we're very guilty of not giving ourselves that and actually feel we are in the wrong by doing that and not giving 100% to children. So I think that's great." [Parent 6]
	Program understanding and confidence	-	"I love the fact… it's very clear what the coaching sessions will look like. That's amazing how you described it and then what topics to discuss. It gives a very clear structure and also us reflecting on our existing skills but also having that goal set mindset to developing new skills and strengthening them." [Clinician 3]
**Feeling understood, supported and connected**	Emotional support and validation	-	"It's just real life here, it's just validating everyday life, everyday things that we all are going through… So the sharing and the validated and normalising is definitely here, I can see that. I like this." [Parent 3]
	Social and peer connection	-	"The fact that you got audio on the first homepage, I love that. And I can see that… real people's story… I can relate… I can see a name, and I can listen to the story so it makes me more interested to see, what's the story? Maybe like me, you know… I really like it." [Parent 4]
**Contextualised and applied knowledge**	Practical knowledge	-	"Especially support with implementation, I think you need that because it's one thing to get the information and everything but it's another thing to put it all into practice." [Parent 1]
	Personalised knowledge	-	"It's so hands-on… giving you specific things to try and giving you options depending on your situation… then having the opportunity to try it and then come back and debrief on it with your counsellor in that space, I think is really wonderful… You can really start to unpack and go into, 'Okay how did this impact you? How did this impact your child? How is it actually playing in with your mental health?" [Clinician 2]
**Parent-led empowerment**	Guided autonomy	-	"That all kind of ties in again with the guided autonomy… the wisdom or experience of the clinician being able to support the parent if they do need help… parents feeling like they're in control but also learning new strategies… it's building confidence." [Clinician 1]
	Parent self-efficacy and self-worth	-	"It's very encouraging. 'Just take one step at a time, be patient, be kind to yourself'. It's all good, it's all positive and solution focused." [Clinical Lead 2]
**Accessible and integrated delivery**	Low-burden accessibility	-	"I do like the language, quite simple, easy there's no complicated words or hard things to understand, which is great... and I like multiple choice rather than open questions." [Parent 2]
	Seamless integration	-	"You are there as a person, and part of you is a parent so absolutely, that would be the space to do it. I don't think you would need to go to a separate place to talk about the parenting component of who you are… But there might be times when you just need the whole session to process something that will then help you the next day in your parenting." [Parent 5]

Supplementary Table S5. Refinements made to the initial prototype and considerations for future iterations alongside Phase 2 themes

Phase 2 Theme	Prototype changes and future considerations
**Building readiness for parenting support**	**Refinements made to the initial prototype during this study:**<br>• Included a crisis helpline number directly on the final page of each module.<br>• Included links for additional mental health resources for parents and children on the 'Useful Resources' page of the online modules.<br>• Added an introductory email to be sent to parents prior to starting the program, explaining the program's purpose and who it is designed for.<br>• Made purpose of reflection questions clearer so parents are introduced to how to use them and their rationale (i.e., helping parents work towards their goals and facilitating support from clinicians or other support people).<br><br>**Considerations for future iterations of the program:**<br>• Quick exit button within the modules for parents facing family violence.<br>• When introducing the program to parents, consider focusing on the individual's mental health by emphasising how strengthening the parent-child relationship can improve parent wellbeing.<br>• Make the purpose of reflection questions in clinician training clearer so clinicians are introduced to the rationale for this program feature.
**Feeling understood, supported and connected**	**Refinements made to the initial prototype during this study:**<br>• More clearly and frequently acknowledged that parenting is hard throughout module content.<br>• Included extra 24/7 helplines within module content to provide parents with emotional support outside of working hours.<br>• Included more lived experience vignettes of other parents with experience of parenting with mental health challenges, including audio and a link to a podcast.<br>• Included additional suggestions for parents to reach out to support network for emotional and practical support. Added reflection question where parents can note when, where and from who they might seek additional support.<br><br>**Considerations for future iterations of the program:**<br>• Create further opportunities for parents to connect with peers e.g., program activities to show a tally of other parent answers, develop an online support group or provide information about local support groups.<br>• Clinicians would like their ongoing supervision to be group based with peers.<br>• Clinicians would like it to be clearer that one purpose of the reflection questions within their training is to share them with peers.
**Contextualised and applied knowledge**	**Refinements made to the initial prototype during this study:**<br>• Included open-ended and tick box fields on several module pages to allow parents space to indicate which strategies/ideas are relevant to them and how they might implement strategies in their life.<br>• Wherever possible, ensured that each parenting strategy in the module content is accompanied by examples of how to practically apply it.<br>• Weaved in additional phrasing throughout modules to consistently contextualise content around parenting while experiencing mental health difficulties.

Phase 2 Theme	Prototype changes and future considerations
	• Ensured the inclusion of examples that may be more applicable to fathers and their parenting preferences e.g., play as well as cuddling for affection.<br>• Ensured module images reflect a balanced representation of different cultures and ethnic backgrounds.<br>• Added a selection of dashboard avatars, allowing parents to choose one that best represents them to personalise their experience.<br>• Ensured that each goal setting section has four to five goals, so parents have more than three goal options to choose from.<br><br>**Considerations for future iterations of the program:**<br>• Incorporate a search function within the modules so parents can quickly locate specific module topics or resources based on their needs.<br>• Consider including realistic images of real people within the modules to help parents see strategies demonstrated in a relatable and practical way.<br>• Weigh up whether to add an open-ended field where parents can record responses to existing prompts (why they chose the goal, what steps they will take), and note which specific activities they chose or whether this would create unnecessary extra work for parents.
**Parent-led empowerment**	**Refinements made to the initial prototype during this study:**<br>• Included open-ended fields on several module pages asking parents to note any other of their own ideas beyond the examples provided.<br>• Made sharing of each module's goals and reflection responses optional, allowing parents control over if/when they share this information with their clinician.<br>• Included encouraging prompts halfway and at the end of each module to affirm parents' efforts, acknowledge progress and provide motivation to keep going.<br>• Revised any language within modules that might unintentionally be deficit-focused or set unrealistic expectations of parents and added extra statements to encourage parents to exercise self-compassion and accept mistakes.<br>• Included opportunities within modules for parents to indicate strategies they already use to encourage them to notice their existing parenting strengths.<br>• Added a progress section on the dashboard of the modules visually showing parents the total number of module pages and modules they have completed.<br>• Added a statement on the 'Check your learning' quiz page reassuring parents that it is okay to answer incorrectly and that this is simply an opportunity for consolidating learning.<br><br>**Considerations for future iterations of the program:**<br>• Weigh up whether to add fully open-ended reflection boxes in each module where parents can note any other comments or reflections. Consider whether this would support parent's independent thinking or create unnecessary extra work.<br>• Consider incorporating a module feature that helps parents track incremental steps towards goals and accumulates a list of completed goals.
**Accessible and integrated delivery**	**Refinements made to the initial prototype during this study:**<br>• Improved readability of module content (e.g., simplified instructions and questions, broke up paragraphs into smaller spaced-out sections or dot points, shortened sentences, made font larger, added varying sizes/colours/bolding to highlight key points).

Phase 2 Theme	Prototype changes and future considerations
	• Added a page at the end of each module to summarise key points.<br>• Reduced frequency of open-text response questions within the modules and included even more multiple choice and tick box formatted questions.<br>• Checked content wording for accessibility and removed any phrasing that could be perceived as clinical, formal or complicated.<br>• Increased interactivity or engaging aspects of the online modules e.g., added more relevant pictures, activities, audio files, text presented in colour-coded boxes, gradual unveiling of text, click to reveal text.<br>• Clinicians to have flexibility to contact parents to check-in with module progress between sessions if they deem this important and within their capacity on a case-by-case basis, but expected commitment outside of sessions remains minimal<br><br>**Considerations for future iterations of the program:**<br>• Consider whether it would be possible to add a time indication to each module or goal while recognising that completion time may vary for each parent or specific activity choice.<br>• Consider the feasibility of adding videos or a text-to-speech function or making the modules available in multiple languages.<br>• Weight up whether to provide parents with a timeframe for completion of the program to encourage finishing or whether this would add unhelpful pressure.<br>• Consider providing parents with instructions on how to download, save or print module content pages.<br>• For future modules where some parenting tips involve a financial cost, make sure to acknowledge financial problems as a potential barrier for parents.<br>• Clinicians would ideally like their ongoing supervision to be run by a supervisor with parenting expertise.
**Bridges for communication**	**Refinements made to the initial prototype during this study:**<br>• Shifted from a design where clinicians have direct dashboard access to parent progress and reflections to parents instead sharing their reflections directly with clinicians via email or in-session.<br>• Incorporated more frequent reminders within module content for parents to seek support from their clinician, strengthening the link between online modules and clinician support.<br><br>**Considerations for future iterations of the program:**<br>• Explore the feasibility of a clinician dashboard that could allow for secure and practical clinician access to parent progress and reflections. If not possible, explore ways to better support parents in independently bringing and sharing this information to their clinicians.<br>• Consider whether to add an open-ended reflection box within module content where parents can note any other questions or information they would like to share with their clinician. Consider whether this would support communication or create unnecessary burden and risk concerns for clinicians, given the potential volume and sensitivity of unstructured reflection.

Supplementary File S2. Selected images of the refined parenting program prototype incorporating features from Phase 1 and 2 findings

Selected images from Module 1:

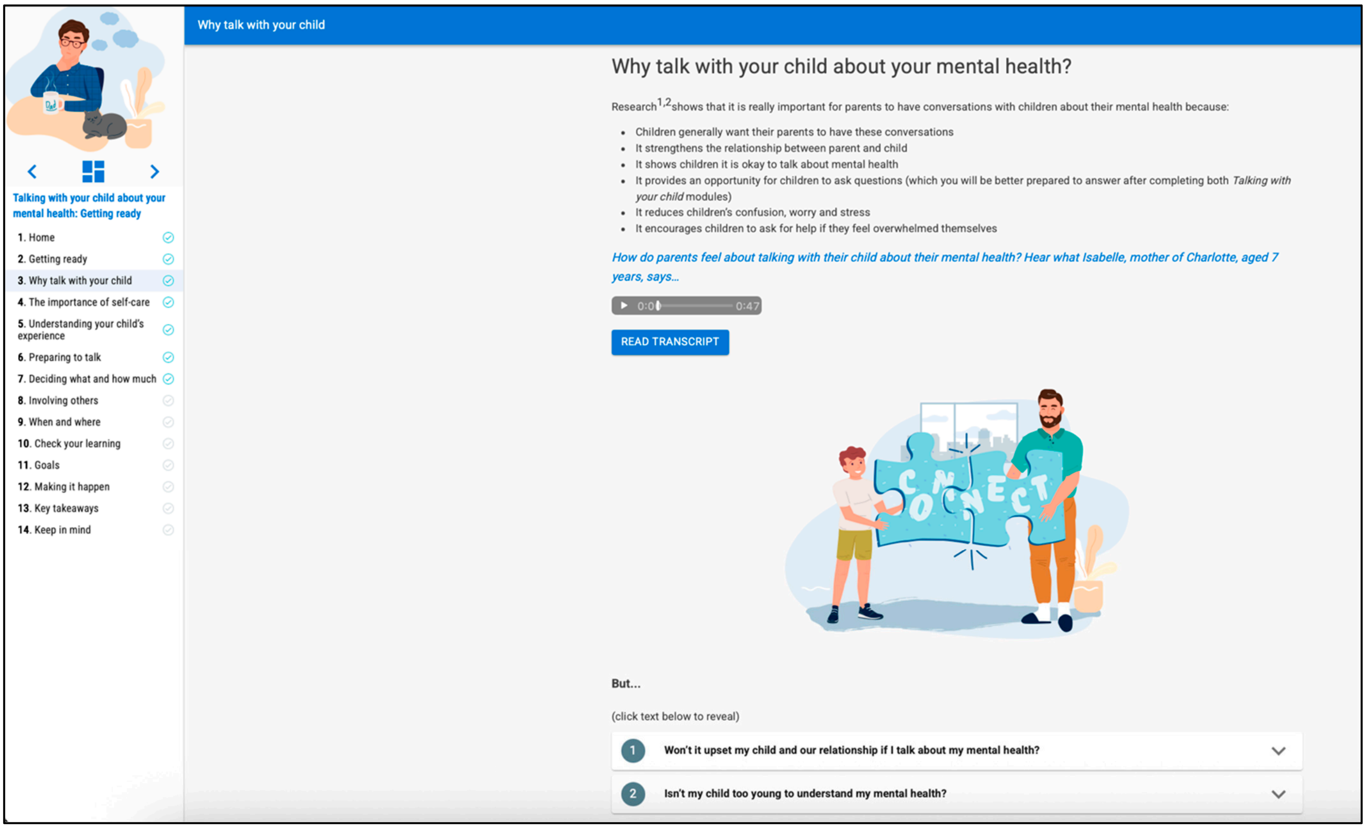

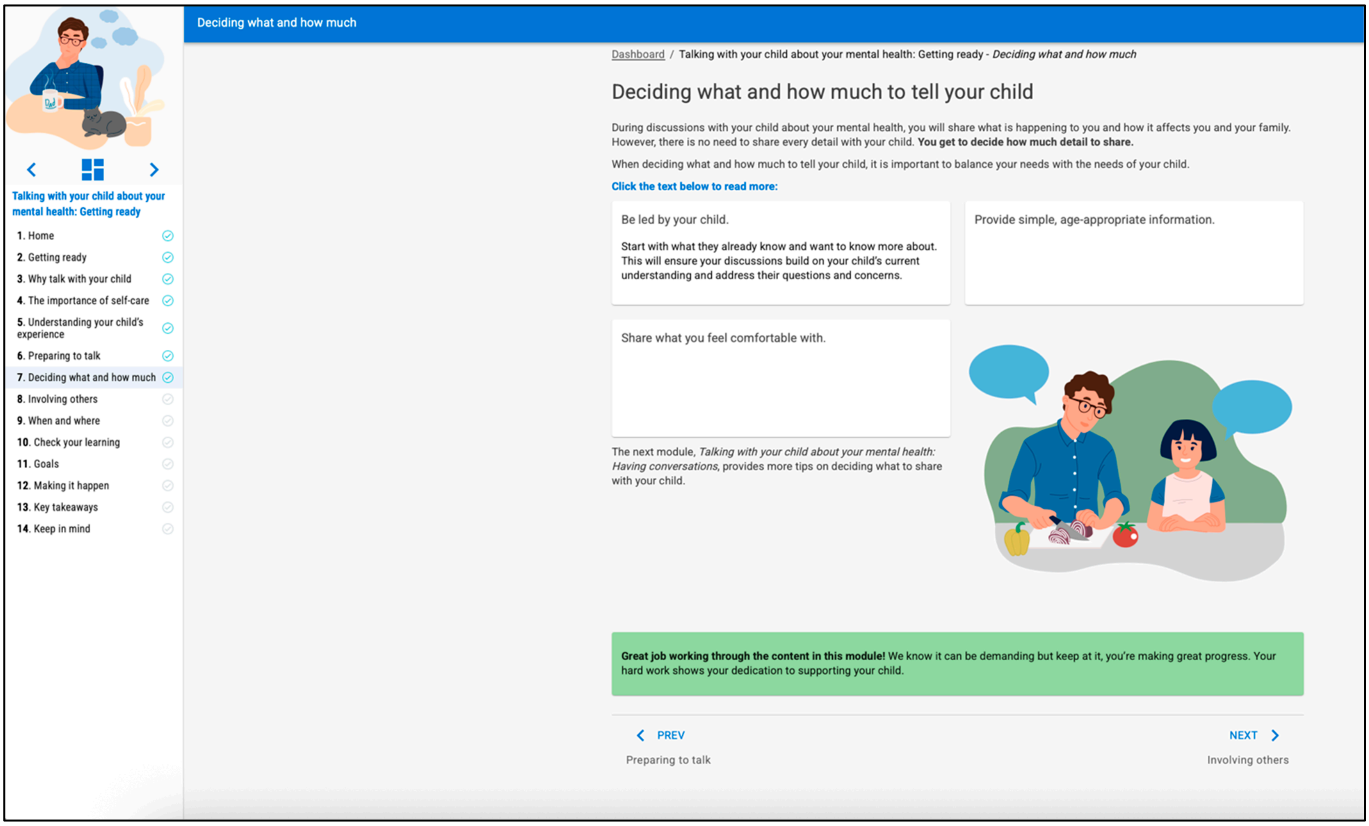

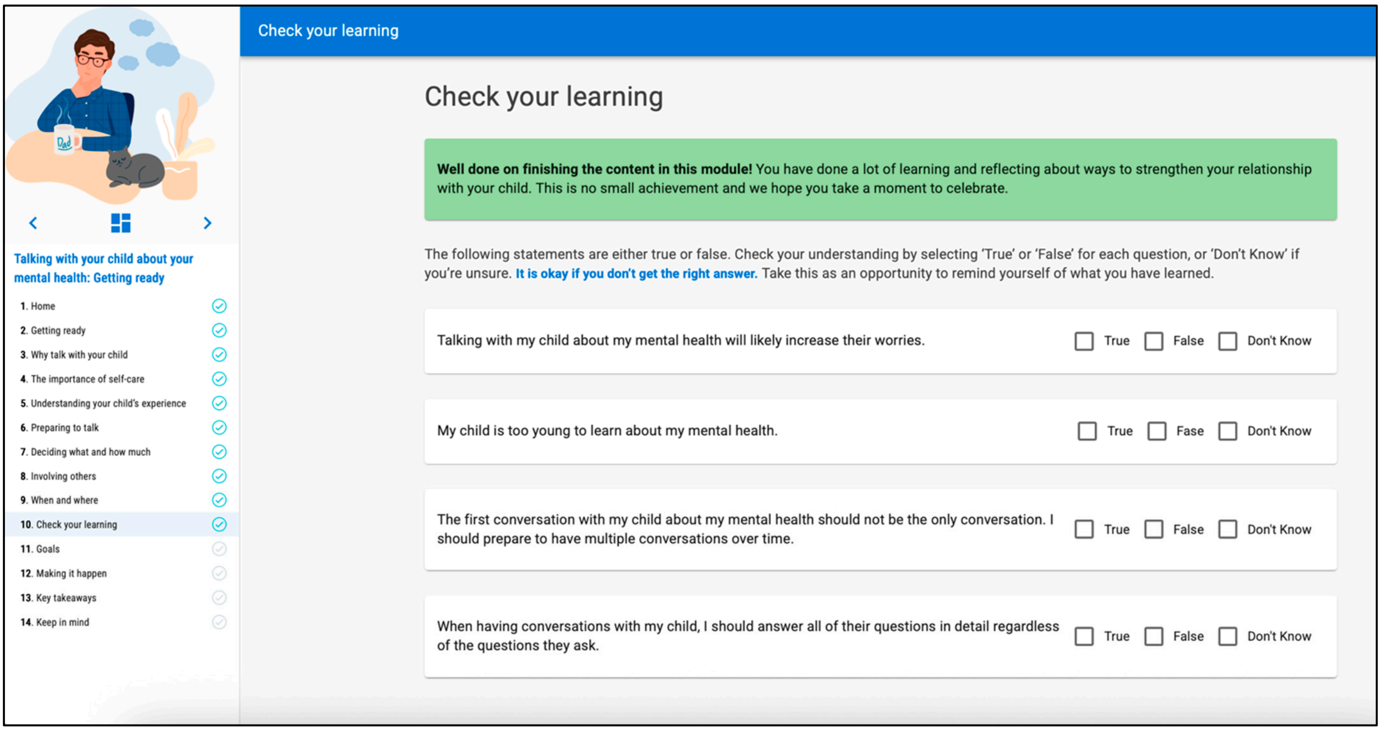
Check your learning
Talking with your child about your mental health: Getting ready
1. Home
2. Getting ready
3. Why talk with your child
4. The importance of self-care
5. Understanding your child's experience
6. Preparing to talk
7. Deciding what and how much
8. Involving others
9. When and where
10. Check your learning
11. Goals
12. Making it happen
13. Key takeaways
14. Keep in mind
Check your learning
Well done on finishing the content in this module! You have done a lot of learning and reflecting about ways to strengthen your relationship with your child. This is no small achievement and we hope you take a moment to celebrate.
The following statements are either true or false. Check your understanding by selecting 'True' or 'False' for each question, or 'Don't Know' if you're unsure. It is okay if you don't get the right answer. Take this as an opportunity to remind yourself of what you have learned.
Talking with my child about my mental health will likely increase their worries.
True False Don't Know
My child is too young to learn about my mental health.
True Fase Don't Know
The first conversation with my child about my mental health should not be the only conversation. I should prepare to have multiple conversations over time.
True False Don't Know
When having conversations with my child, I should answer all of their questions in detail regardless of the questions they ask.
True False Don't Know

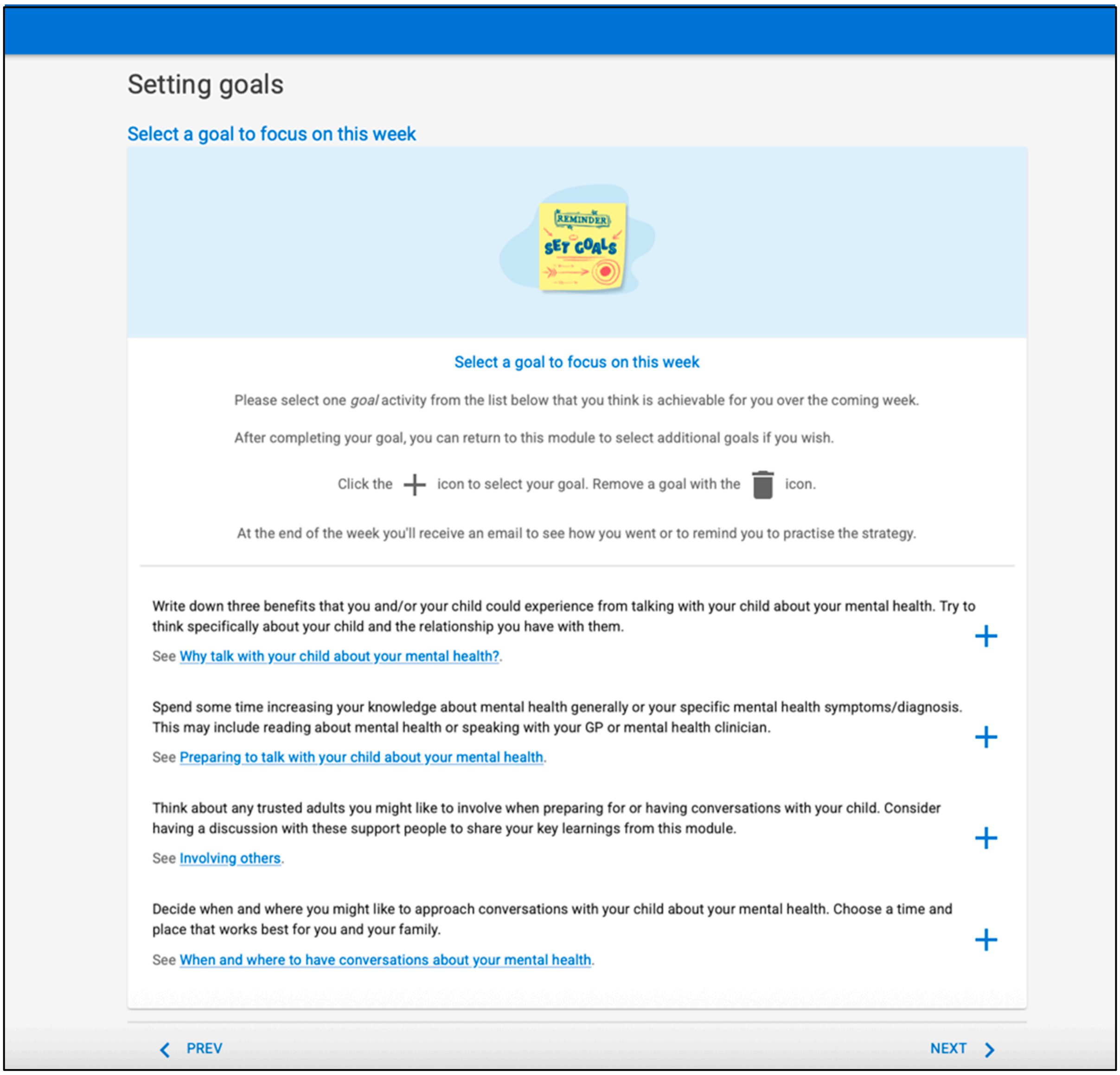
Setting goals
Select a goal to focus on this week
REMINDER
SET GOALS
Select a goal to focus on this week
Please select one goal activity from the list below that you think is achievable for you over the coming week.
After completing your goal, you can return to this module to select additional goals if you wish.
Click the + icon to select your goal. Remove a goal with the icon.
At the end of the week you'll receive an email to see how you went or to remind you to practise the strategy.
Write down three benefits that you and/or your child could experience from talking with your child about your mental health. Try to think specifically about your child and the relationship you have with them.
See Why talk with your child about your mental health?.
Spend some time increasing your knowledge about mental health generally or your specific mental health symptoms/diagnosis. This may include reading about mental health or speaking with your GP or mental health clinician.
See Preparing to talk with your child about your mental health.
Think about any trusted adults you might like to involve when preparing for or having conversations with your child. Consider having a discussion with these support people to share your key learnings from this module.
See Involving others.
Decide when and where you might like to approach conversations with your child about your mental health. Choose a time and place that works best for you and your family.
See When and where to have conversations about your mental health.
PREV
NEXT

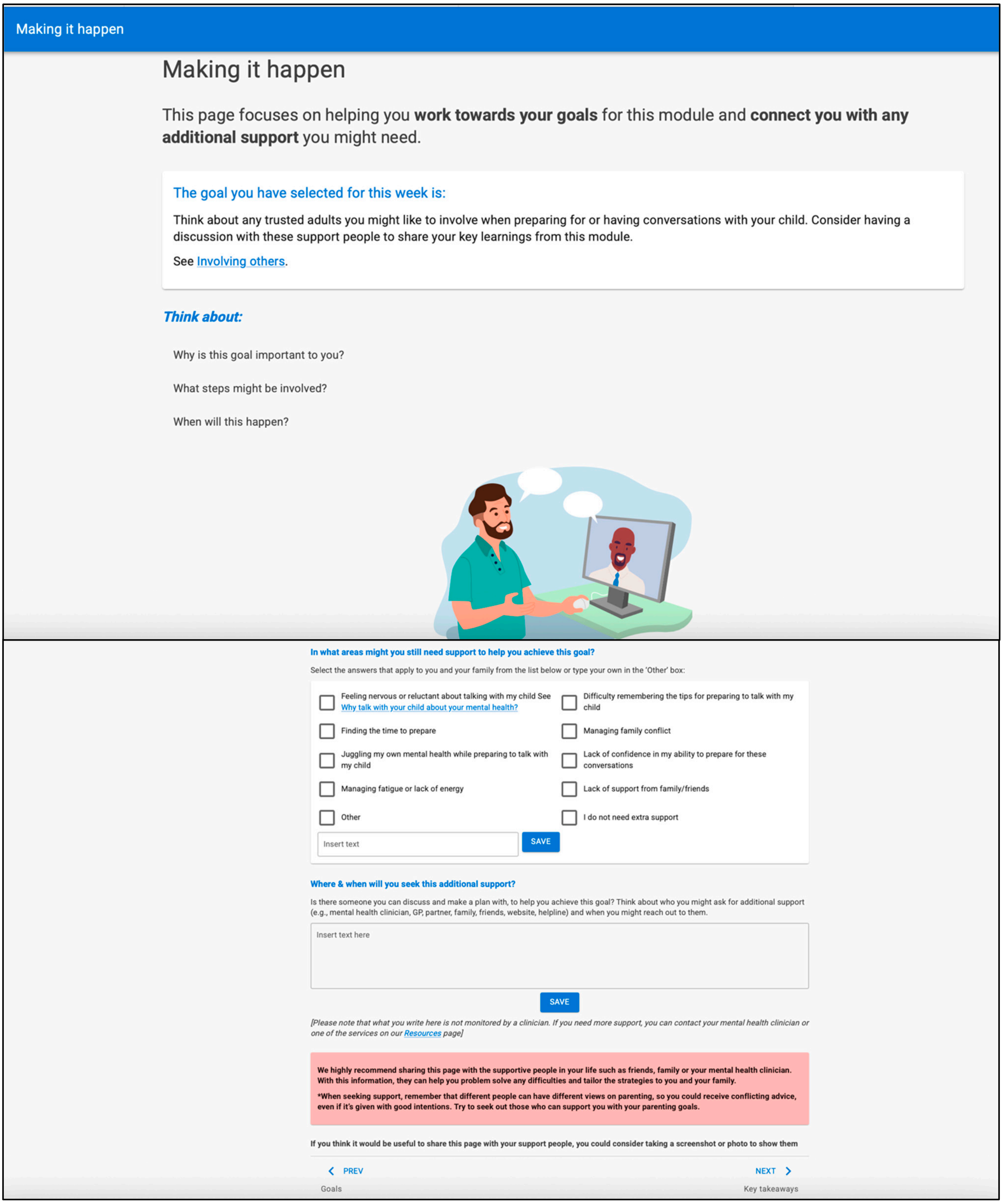
Making it happen
Making it happen
This page focuses on helping you work towards your goals for this module and connect you with any additional support you might need.
The goal you have selected for this week is:
Think about any trusted adults you might like to involve when preparing for or having conversations with your child. Consider having a discussion with these support people to share your key learnings from this module.
See Involving others.
Think about:
Why is this goal important to you?
What steps might be involved?
When will this happen?
In what areas might you still need support to help you achieve this goal?
Select the answers that apply to you and your family from the list below or type your own in the 'Other' box:
Feeling nervous or reluctant about talking with my child See Why talk with your child about your mental health?
Difficulty remembering the tips for preparing to talk with my child
Finding the time to prepare
Managing family conflict
Juggling my own mental health while preparing to talk with my child
Lack of confidence in my ability to prepare for these conversations
Managing fatigue or lack of energy
Lack of support from family/friends
Other
I do not need extra support
Insert text
SAVE
Where & when will you seek this additional support?
Is there someone you can discuss and make a plan with, to help you achieve this goal? Think about who you might ask for additional support (e.g., mental health clinician, GP, partner, family, friends, website, helpline) and when you might reach out to them.
Insert text here
SAVE
[Please note that what you write here is not monitored by a clinician. If you need more support, you can contact your mental health clinician or one of the services on our Resources page]
We highly recommend sharing this page with the supportive people in your life such as friends, family or your mental health clinician. With this information, they can help you problem solve any difficulties and tailor the strategies to you and your family.
*When seeking support, remember that different people can have different views on parenting, so you could receive conflicting advice, even if it's given with good intentions. Try to seek out those who can support you with your parenting goals.
If you think it would be useful to share this page with your support people, you could consider taking a screenshot or photo to show them
PREV
Goals
NEXT
Key takeaways

Selected images from Module 2:

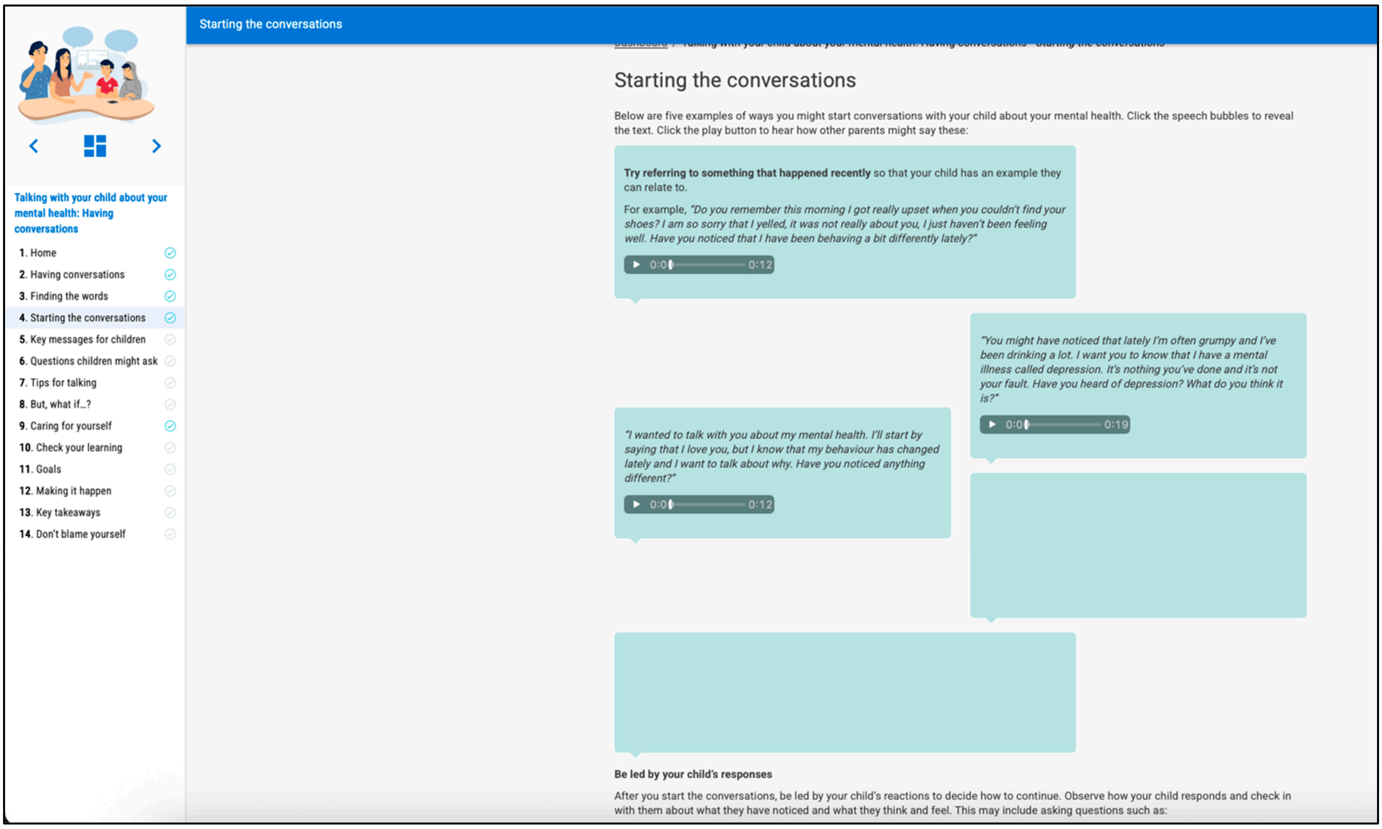

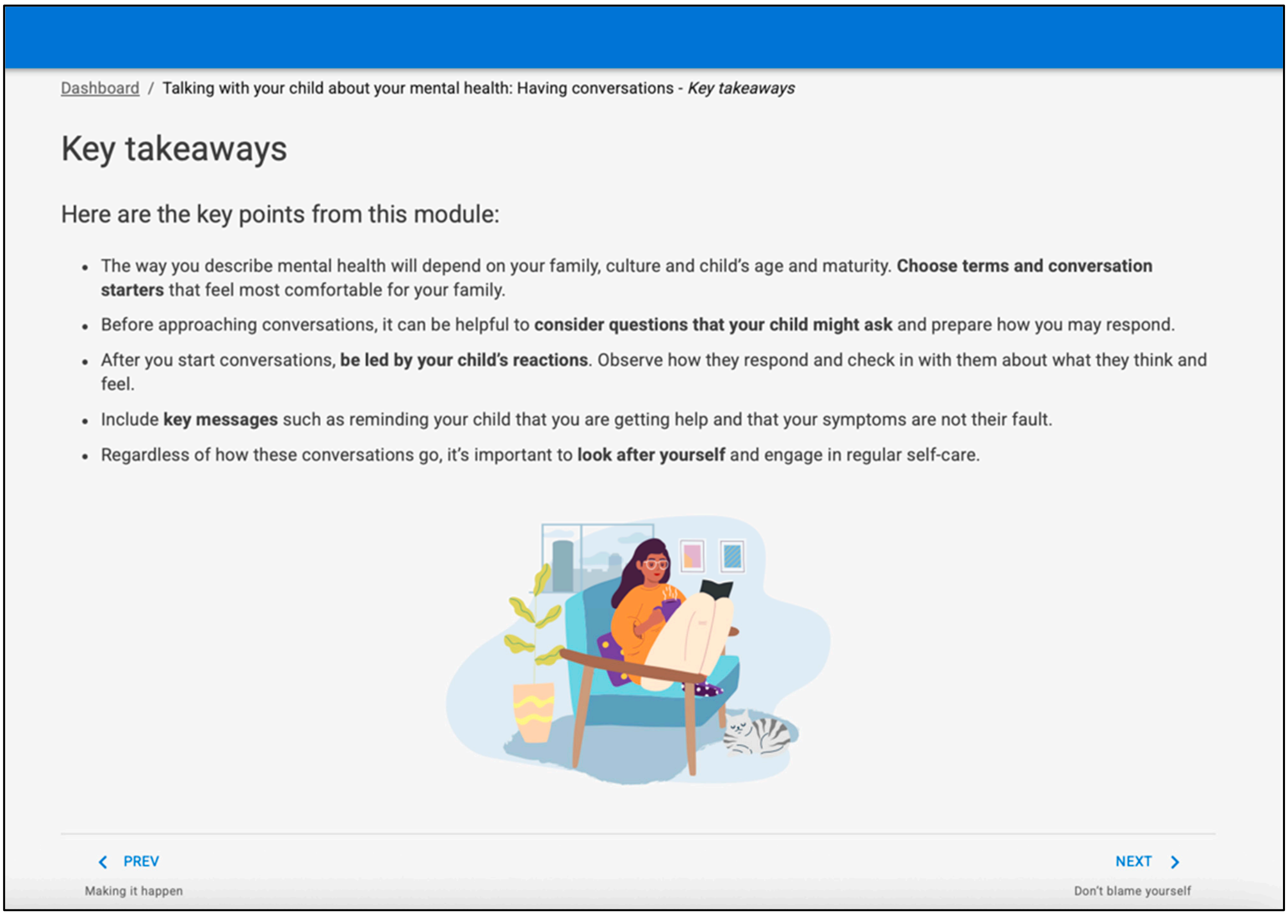

Using co-design to adapt a digital parenting program for parents seeking mental health support

Selected images from Module 3:

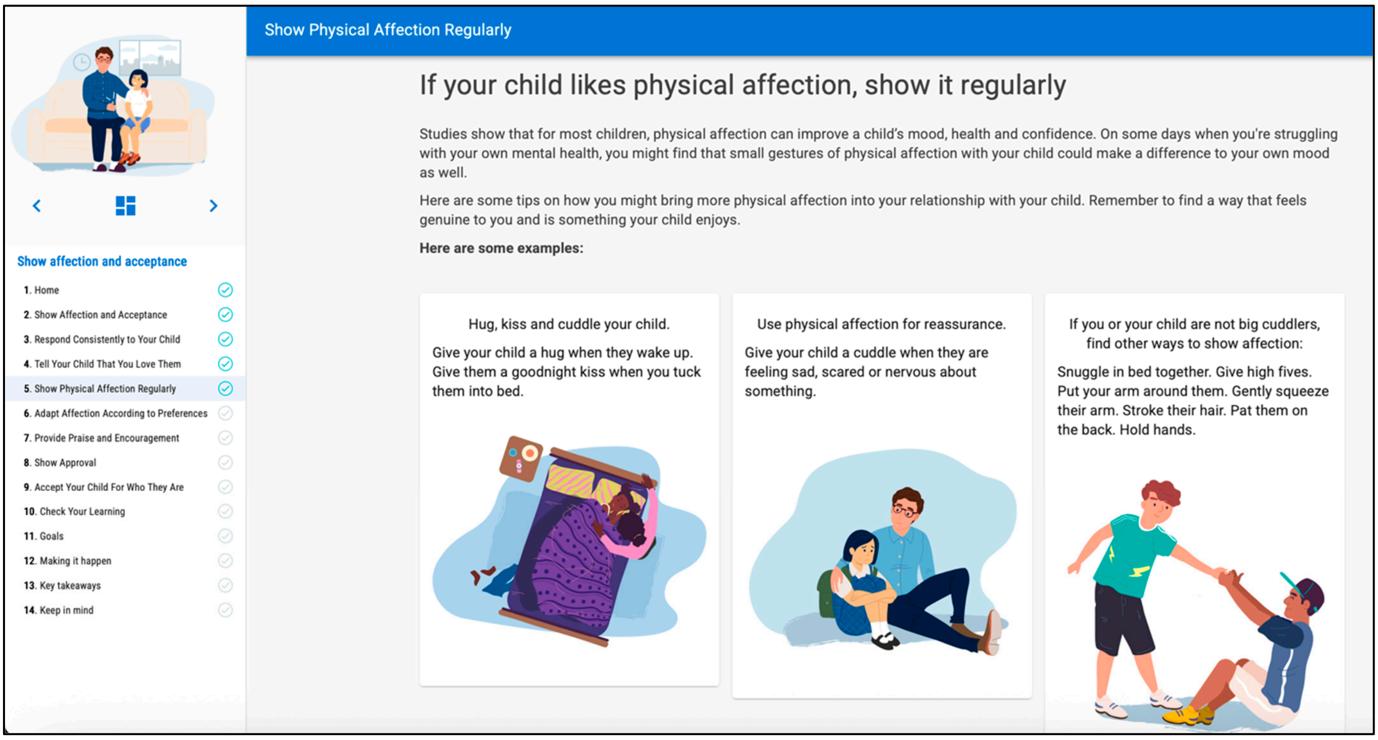

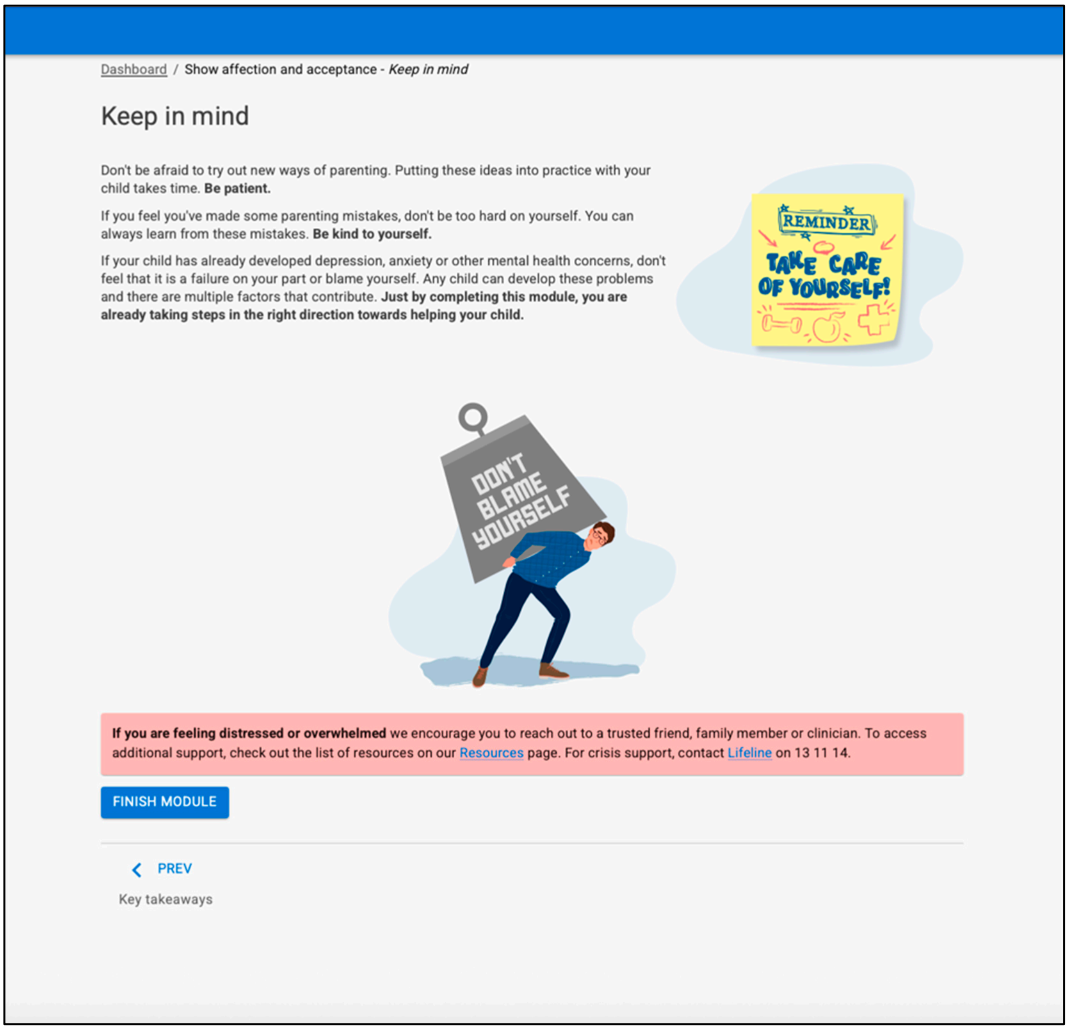

Using co-design to adapt a digital parenting program for parents seeking mental health support

Supplementary Table S6. Demographics of Phase 3 parent participants

Demographics of parents who completed the refined prototype and interview (N = 3)

Demographic characteristic	Value
**Age in years, mean (SD; range)**	47 (7.94; 41-56)
**Gender, n (%)**	
Women	3 (100%)
**Parent relationship to child, n (%)**	
Mother	2 (66.7%)
Grandmother	1 (33.3%)
**Ethnic/Racial background, n (%) ***	
Australian / New Zealand	2 (66.7%)
East Asian (e.g., China, Japan, Korea)	1 (33.3%)
Middle Eastern (e.g., Turkey, Iraq, Iran)	1 (33.3%)
**Parent self-reported mental health challenges, n (%) ***	
High stress	3 (100%)
Anxiety	2 (66.7%)
Trauma or posttraumatic stress disorder (PTSD)	2 (66.7%)
Depression	1 (33.3%)
**Time since last psychological/counselling support**	
Currently accessing support (< 1 month)	2 (66.7%)
3 - 4 months ago	1 (33.3%)
**Number of children aged 5-11 years, n (%)** [1]	
1	2 (66.7%)
2	1 (33.3%)
**Highest education level, n (%)**	
Technical and further education certificate (TAFE) or other technical qualification	1 (33.3%)
Bachelor's degree	1 (33.3%)
Postgraduate	1 (33.3%)
**Concession hard holder, n (%)** [2]	3 (100%)
**Lives without another adult at home, n (%)**	3 (100%)
**State of residence, n (%)**	
Victoria	1 (33.3%)
South Australia	2 (66.7%)

[1] Refers to children aged 5-11 years as the grandmother participant also had two adult children.

[2] Low-income earners and/or recipients of government benefit schemes with access to discounted prescription medicines, healthcare, public transport and other government services.

* *Note:* Parents could select more than one ethnic/racial background or mental health issue; these percentages do not sum to 100%.

Using co-design to adapt a digital parenting program for parents seeking mental health support

Supplementary File S3. Phase 3 parent interview schedule

Research Aim: Evaluate the co-designed prototype intervention with parents to understand its acceptability and what, if any further adaptations are needed.

NOTES

- Acceptability interview questions will be based on items from the Theoretical Framework of Acceptability (TFA) questionnaire (Sekhon et al., 2022). The conversation will be semi-structured so questions are a guide only and not all questions will be asked to every parent.
- As a general rule, probe for more information until you are confident that the participant has said all that they wish to on that topic.

INTRODUCTION

- Thank parent for attending
- Remind parent of purpose of interview:
  - The purpose of this feedback chat is to hear their feedback on the parenting program they recently completed.
  - Explain that we intend this program to eventually become part of an existing mental health service for parents seeking mental health support. We are hoping to understand what they think about that too.
  - By understanding this, we're hoping to make some changes to the program where possible.

- Explain that both positive and negative feedback is important and welcomed.
- Explain that we have some set questions to ask but also that we would like to hear any open feedback too.
- Explain that the interview will take around 60 minutes with flexibility for it to be shorter or longer depending on how much feedback the parent has.
- Remind parent that they can change their mind and stop at any time.

- Remind about recording. Explain that:
  - We are recording the interview so that we can transcribe what is said and have an accurate record.
  - The recording will be stored securely and only accessible by the research team.
  - The recording will be stored separately from any personal details about them (e.g., name and contact details).

- Ask if they have any questions or concerns.

Using co-design to adapt a digital parenting program for parents seeking mental health support

START RECORDING

Topic	Central Questions	Probes/Prompts
**General acceptability**<br><br>**TFA construct: Affective attitude**<br>How an individual feels about the intervention<br><br>"Did you like or dislike [intervention]"<br>OR<br>"How comfortable did you feel [to engage with] [intervention]"	"To start with, it would be great to hear about your overall impressions / feedback of the program."<br><br>1. Overall, what did you think of the program?<br><br>2. How would you describe your overall experience with the program?<br><br>3. Overall, did you like or dislike the program?	- What, if anything, did you like about the program?<br>- What, if anything, did you like the least or dislike about the program?<br><br>- Was anything missing?<br>- What do you think could be changed?<br>- How could the program be adapted to overcome or improve [X]?<br><br>- Did you experience discomfort of any kind as a result of engaging with the program?
**TFA construct: Intervention coherence**<br>"The extent to which the participant understands the intervention and how it works (i.e. the 'face validity' of the intervention for the recipient)"	- How similar was the program to what you expected?	- How so?<br>- Why is that?
**TFA construct: Perceived effectiveness**<br>"The [intervention] has improved [behaviour/ condition/clinical outcome]"	What, if any, were some of the biggest take-aways from the modules for you?<br><br>Have you made any changes to your parenting based on the content during or after the program?<br>--------------------------------<br>The program was designed to support parents seeking mental health support:<br>- How helpful was the program, if at all, in understanding, validating and normalising your experiences as a parent?<br>- Did the program increase or decrease your confidence, belief in yourself or knowledge of your strengths?	- If yes, tell me about them, and what you think helped bring about that change.<br>- If no, why not? What stood in the way?<br><br>--------------------------------<br>How much did the program help you with doing and applying what you learned about parenting in your everyday life?

Topic	Central Questions	Probes/Prompts
**TFA construct: Ethicality**<br>The extent to which the intervention has good fit with an individual's value system.<br><br>"How fair is [intervention] for [recipients] with [condition]?" "There are moral or ethical consequences to engage with [intervention]"	How appropriate/tailored was the program to your personal needs and your family context?<br><br>*For example,* who is in your family, your cultural or religious background, your financial position or education, your values towards parenting, and your mental health concerns.	- How well did you feel your family context was represented in the program?<br><br>- How tailored, relevant and flexible was the program to your mental health concerns?<br>- Did you feel excluded or offended by any aspects of the program?<br>- Tell me about a time where it felt tailored, and/or a time where it did not feel tailored.
**TFA construct: Burden**<br>The amount of effort required to participate in the intervention.<br><br>"How much effort did it take [to engage in] [intervention]?	How much effort did it take for you to do the program?<br><br>Did you encounter any difficulties completing the modules?	- How did you feel about the amount of effort required of you to do the program?
**TFA construct: Opportunity costs**<br>The benefits, profits or values that were given up to engage in the intervention.<br><br>"[Engaging with] [intervention] interfered with my other priorities"	What, if anything, did you need to give up or sacrifice in order to do the program?	- How did you feel about having to give up/sacrifice those things in order to do the program?
**TFA construct: Self-efficacy**<br>The participants' confidence that they can perform behaviour(s) required to participate in the intervention<br><br>"How confident did you feel about [engaging with] [intervention]?"	How confident did you feel about your ability to complete the program?<br><br>- Was the program achievable and realistic? Why/why not?	How confident did you feel in your ability to…<br>- do the online modules?<br>- complete your goals?<br>- make adjustments to your parenting?<br>- identify areas of support and access additional support?
Integration of a clinician-support component	You've completed the program as a stand-alone online program that you did in your own time. I'd like you to imagine the program was being offered to you by your mental health clinician. In this situation you would still complete the modules but could ask for support from	- Would you like to be able to discuss what you learnt in this parenting program with your clinician during your usual sessions? Why/Why not?<br><br>- In this scenario, what (if anything) would help you to complete the program and talk about it with your clinician?

Topic	Central Questions	Probes/Prompts
	your existing mental health clinician as part of your usual counselling sessions. What are your thoughts on this?<br><br>What do you like / not like about self-guided online modules paired with clinician support?	- What kinds of things would make it difficult to complete the program and talk about it with your clinician?
**Other suggestions**	Do you have any other comments or suggestions for the program that we haven't discussed so far?<br><br>Do you have any questions or ideas you want to bring up to the team behind this program?	**General probes**<br>- Can you tell me more?<br>- How so? In what ways?<br>- Why / why is that / why was that the case for you?<br>- Why is that important to you? Why not?<br>- Could you give me an example?

General probes
- Can you tell me more about that?
- How so?
- In what ways?
- Why / why is that / why was that the case for you?
- Why is that important to you? Why not?
- Could you give me an example?

CONCLUSION

- Conclude and summarise key insights
- Remind parent that if the interview has brought up any distress to seek support from family, friends, health professionals or helplines as appropriate
- Explain about member checking of transcripts and ask if parent would like to review their transcript
- Remind about reimbursement
- Thank parent for sharing opinions and time
